# Comparative transcriptomic analysis of roots of contrasting *Gossypium herbaceum* genotypes revealing adaptation to drought

**DOI:** 10.1186/1471-2164-13-680

**Published:** 2012-11-29

**Authors:** Alok Ranjan, Neha Pandey, Deepika Lakhwani, Neeraj Kumar Dubey, Uday V Pathre, Samir V Sawant

**Affiliations:** 1 CSIR-, National Botanical Research Institute, Rana Pratap Marg, Lucknow, 226001, India

**Keywords:** *G.herbaceum* genotypes, Root length, Osmotic stress, Microarray, Pyrosequencing, Gene expression

## Abstract

**Background:**

Root length and its architecture govern the adaptability of plants to various stress conditions, including drought stress. Genetic variations in root growth, length, and architecture are genotypes dependent. In this study, we compared the drought-induced transcriptome of four genotypes of *Gossypium herbaceum* that differed in their drought tolerance adaptability. Three different methodologies, namely, microarray, pyrosequencing, and qRT–PCR, were used for transcriptome analysis and validation.

**Results:**

The variations in root length and growth were found among four genotypes of *G.herbaceum* when exposed to mannitol-induced osmotic stress. Under osmotic stress, the drought tolerant genotypes Vagad and GujCot-21 showed a longer root length than did by drought sensitive RAHS-14 and RAHS-IPS-187. Further, the gene expression patterns in the root tissue of all genotypes were analyzed. We obtained a total of 794 differentially expressed genes by microarray and 104928 high-quality reads representing 53195 unigenes from the root transcriptome. The Vagad and GujCot-21 respond to water stress by inducing various genes and pathways such as response to stresses, response to water deprivation, and flavonoid pathways. Some key regulatory genes involved in abiotic stress such as AP2 EREBP, MYB, WRKY, ERF, ERD9, and LEA were highly expressed in Vagad and GujCot-21. The genes RHD3, NAP1, LBD, and transcription factor WRKY75, known for root development under various stress conditions, were expressed specifically in Vagad and GujCot-21. The genes related to peroxidases, transporters, cell wall-modifying enzymes, and compatible solutes (amino acids, amino sugars, betaine, sugars, or sugar alcohols) were also highly expressed in Vagad and Gujcot-21.

**Conclusion:**

Our analysis highlights changes in the expression pattern of genes and depicts a small but highly specific set of drought responsive genes induced in response to drought stress. Some of these genes were very likely to be involved in drought stress signaling and adaptation, such as transmembrane nitrate transporter, alcohol dehydrogenase, pyruvate decarboxylase, sucrose synthase, and LEA. These results might serve as the basis for an in-depth genomics study of *Gossypium herbaceum*, including a comparative transcriptome analysis and the selection of genes for root traits and drought tolerance.

## Background

During this century, water limitations are likely to increase in agriculture, and hence, the development of crop varieties with better water use efficiency (WUE) is of prime importance in agriculture. The root significantly contributes to the management of water stress and the adaptability of plants to stress [1]. Thus, recently, a significant number of studies focusing on genetic variation in root development and its response to drought and other abiotic stress were reported in wheat [1], soybean [2], and maize [3,4]. Root length depicts the adaptability for drought stress in *Oryza sativa*[5,6]. Larger root systems provide the ability for plants to compete for nutrients, give them support for water uptake, and enable them to survive in water deficiency [7,8]. Root system development and its architectures is determined by intrinsic genetic properties; modulated by various environmental factors, nutrients, and water availability; and presumably governed by many genes [9]. Several genes that control root architecture and development have been identified in *Arabidopsis thaliana*, *Zea mays*, and *Oryza sativa* in recent genetic studies [10-12]. The list of genes includes LBD16/ASL18, LBD29/ASL16, and CRL1/ARL1, which are reported to regulate lateral root formation and help in root gravitropism [10]. Further, RHD3 was reported to be responsible for the formation and elongation of root hairs [13]. The transcription factors HD-ZIP and PLETHORA (PLT) were primarily responsible for the molecular origin of the root and cell-type differentiation [14,15]. Similarly, other transcription factors, namely WRKY75, ZAT6 [16-18], and bHLH32 [19], have been reported to play a critical role in root development and growth under phosphate deficiency. The root plays a vital role in maintaining the physiological condition of plants during water stress. The transpiration rate and stomatal conductance of plants are reduced during drought stress, and they are stimulated by chemical and hormonal signaling before hydraulic signaling in the roots [20]. Various signaling molecules such as auxin and cytokinin are produced in the roots and play a crucial role in shoots during the drought stress in plants [21]. The auxin helps in cell division, tropisms, vascular differentiation, and maintenance of the root meristem [22]. Simultaneously, in response to external stimuli such as drought, it changes the transcriptional programs of specific cells types and transmits the spatial information in different plant organs [23]. Cytokinin signaling is mediated through histidine kinase pathways in root tissues under the drought condition [24]. Cytokinin regulates the pattern formation and differentiation of the vascular tissue of roots during root development [25]. Various signal transduction cascades mediating hormonal signaling in response to nutrient deprivation and drought stress and their transcriptional responses have been elucidated from microarray and transcriptome sequencing studies [26]. Drought stress creates a redox imbalance in plant cells, resulting in a cascade of changes in various signaling pathways, cell wall-membrane integrity, and produces various antioxidant enzymes [27]. Many of these enzymes, including superoxide dismutases (SODs), ascorbate peroxidases (APXs), catalases (CATs), glutathione peroxidases, and peroxiredoxins, help in the maintenance of the redox balance of cells under the stress condition [28]. The roles of various membrane transporters have been characterized under drought and salt stress, which provide osmotic balance and ionic homeostasis in plant cells [29].

The diploid (2n = 26) species of cotton, namely G*. herbaceum* and *G.arboreum*, are indigenous in Asia and Africa and are commonly referred to as desi cottons in India [30,31]. The *Gossypium herbaceum* (A1-genome) and *Gossypium arboreum* (A2-genome) have an inherent ability to sustain drought stress [32]. In drought-prone areas in Asia, these two species are preferentially cultivated due to their ability to withstand drought [32]. We recently published a detailed physiological investigation and conducted genome-wide expression studies on leaves of drought-tolerant Vagad and drought-sensitive RAHS-14 genotypes [31]. We identified the various genes and inherent biochemical pathways that interact in drought-tolerant genotypes to provide them with an adaptive advantage in the stress condition [31]. Here, we report the expression profiling of the root of four genotypes of *Gossypium herbaceum*, namely Vagad, GujCot-21 (drought tolerant), RAHS-14, and RAHS-IPS-187 (drought sensitive) using Affymetrix cotton expression array and GS-FLX pyrosequencing in response to drought stress. We identified many stress-responsive genes and biochemical pathways that were differentially expressed in roots during the drought condition in the selected genotypes. Further, we could assign these root-specific genes to different root zones based on the Genevestigator tool. These differentially expressed, tissue-specific genes in the contrasting genotypes reflect the probable mechanistic details of the adaptability of tolerant genotypes to water stress.

## Methods

### Measurement of root length

Cotton (*G.herbaceum*) seeds of Vagad, GujCot-21, RAHS-IPS-187, and RAHS-14 were sterilized, aseptically immersed in water for a day at 30°C, and then placed for germination in a moist petri dish under the following condition: 28°C/25°C as day and night temperature, 12 h of light and dark period alternatively, and a relative humidity of 80%. After 3 days, the properly germinated seeds were transferred to paper rafts, which were submerged (3/4^th^) in Hoagland’s media containing a different percentage of mannitol (4%, 6%, and 8%), and only Hoagland’s media was considered as a control [31]. The seedlings were then allowed to grow until they displayed healthy growth in the control condition; next, the root lengths of the grown seedlings of all genotypes were photographed and measured.

### Drought treatments and growth condition

Cotton (*G.herbaceum*) seeds of four genotypes, namely Vagad, RAHS-14, GujCot-21, and RAHS-IPS-187, were germinated with six seedlings each in an earthen pot in the soil (one plant/pot) and grown in a greenhouse (16-h-light/8-h-dark cycles) at 28°C to 30°C with a relative humidity of 50-60% and a photosynthetically active radiation of 900 μmol m-^2^ s-^1^. After 4 weeks, plants containing six leaves were selected for the experiments and drought stress was imposed by withholding water for seven days (soil moisture below 30%), and control plants were watered regularly [31]. After 7 days of drought treatment, drooping effects on plant leaves became prominent; the plants were uprooted, and the roots of both control and drought treated plants were homogenized in liquid nitrogen before proceeding with RNA isolation.

### Isolation of RNA and real-time PCR

Total RNA was isolated in three independent biological replicates of each genotype using the Spectrum Plant Total RNA Kit (Sigma-Aldrich) according to the manufacturer’s instructions and eluted with nuclease-free water. After DNaseI (Ambion) treatment, they were quantified, and checked for integrity using a Bioanalyzer (Agilent, Inc., Palo Alto, CA, USA). For the analysis of target genes by RT-PCR, cDNA was synthesized from the total RNA of Vagad and RAHS-14 by reverse transcriptase using the cDNA synthesis kit (Invitrogen) according to the manufacturer’s instructions. The RT-PCR was performed in replicates with 1 μl cDNA using the SYBR Green Master Mix (Applied Biosystems) with an ABI 7500 sequence detection system as prescribed in the manufacturer’s protocol (Applied Biosystems). The gene-specific and ubiquitin (internal control) primers were designed using PRIMER3 (http://frodo.wi.mit.edu/primer3/input.htm). The primer details are listed in Additional file 1. The relative quantification method (ΔΔCT) was used for the quantitative evaluation of the gene expression.

### Affymetrix gene chip experiment and microarray data analysis

Three sets of biological replicates of Vagad and RAHS-14 under control and drought conditions (3x2x2) were taken independently, constituting a total of twelve cotton chips for analysis. For Affymetrix gene chip analysis (Affymetrix, Santa Clara, CA, USA), 1 μg total RNA of root tissue was used for making biotin-labeled cRNA targets, hybridization. Synthesis of cDNA, cRNA, and its fragmentation, hybridization, washing, staining, and scanning were conducted according to the gene chip standard protocol (Eukaryotic Target Preparation; Affymetrix). The signal intensity of each probe set on the cotton gene chip was analyzed using Affymetrix GCOS software, and the target mean value (TGT) was scaled as being 500 for each chip. Student’s t-test analysis and log_2_-transformed signal ratio of each probe set were carried out by the Array Assist Software 5.2.2 (Agilent Technologies, Santa Clara, CA, USA). Differentially expressed genes with a detection p value ≤ 0.05 were considered present in three biological replicate experiments. Gene expression data analyses were completed using a filtered RMA expression value. The annotation of the probe set in the Affymetrix cotton genome array was mapped with the locus ID of Arabidopsis TAIR10 version by BLAST. Based on the annotation, the expressed genes were analyzed. Gene ontology analysis was performed for the functional categorization of differentially expressed genes using agriGO tool (http://bioinfo.cau.edu.cn/agriGO/), and the p-values were corrected by applying the false discovery rate correction to control falsely rejected hypothesis during the analysis. The FDR corrected p-value of ≤0.05 was assumed as the cutoff value. Microarray gene expression data used in this study were MIAME compliant and deposited in NCBI-GEO with accession number GSE36249.

### Double strand cDNA library construction and GS-FLX pyrosequencing

Total RNA (3 μg) from the root tissue of GujCot-21 and RAHS-IPS-187 was reverse transcribed using a T7-Oligo (dT) Promoter Primer in the first-strand cDNA synthesis (Affymetrix). After RNase H-mediated second-strand cDNA synthesis, the double-stranded cDNA was enriched and served as a template in the subsequent *in vitro* transcription (IVT) reaction (Affymetrix). The IVT reaction was carried out in the presence of T7 RNA Polymerase (Affymetrix). The cRNA (3 μg) was reverse transcribed in the first-strand cDNA synthesis step by using a random hexamer primer, followed by RNase H-mediated second-strand cDNA synthesis in replicates. The replicate samples were pooled and purified by a column (QIAquick PCR purification kit) and further used for GS-FLX pyrosequencing.

### Emulsion based clonal amplification

Double-strand cDNAs obtained from Gujcot-21 and RAHS-IPS-187 were used for GS FLX library preparation. Approximately 5 μg of double-strand cDNA was sheared by nebulization at 206 kPa for 2–4 min. The fragmented cDNA were amplified in aqueous droplets that were made through the creation of a PCR reaction mixture in emulsion oil (Roche Diagnostics). The droplets acted as separate microreactors in which parallel DNA amplifications were performed, yielding approximately 10^7^ copies of a template per bead. After PCR, the emulsion was broken to release the beads containing the amplified DNA template. The beads carrying the templates were enriched and deposited by centrifugation into the open wells of a 70 × 70 mm^2^ optical picotiter plate for sequencing.

### Assembly and annotation of transcriptome

All sequence analyses were conducted using a publicly available software, R package (http://www.R.project.org). The pyrosequencing reads were assembled after quality control using software version 2.5 newbler, and the assembly was performed using a 40 bp overlap length and a 90% identity. Transcripts annotation was performed by BLASTX using the Basic Local Alignment Tool against the NR database from NCBI (http://www.ncbi.nlm.nih.gov/); from TAIR (http://www.arabidopsis.org/) and the BLASTN for EST cotton database available at NCBI. The GS-FLX sequence reads discussed in this article can be found in the Genebank (http://www.ncbi.nlm.nih.gov/genbank) of the National Center for Biotechnology with accession number SRA029162.1.

### Digital expression analysis

For the digital expression analysis, the reads for both libraries (GujCot-21 and RAHS-IPS-187) were tagged and pooled to form a large dataset of 104928 reads. These reads were assembled using the newbler assembler at an overlapping of 40 bp and a 90% identity. These reads were assembled into 2080 contigs. We calculated TPM value and R value using the R statistics for supercontigs and and those with R value ≥ 3 and Fold change ≥ 2 were considered significantly differentially expressed contigs [33]. These filtered contigs were annotated using BLASTX against the NCBI-NR database, the TAIR, and BLASTN was used for the cotton EST public database. The Genevestigator (https://www.genevestigator.com) software was used for the tissue-specific expression analysis of differentially expressed genes.

### GO and KOBAS analysis

The functional classification of unigenes was performed by assigning gene ontology annotation codes. Only those unigenes that expressed a significant Blast result against the TAIR database were used for GO annotation. The GO annotation for level three was extracted for each library and used for further analysis. The biochemical pathway assignments were carried out according to kobas (http://kobas.cbi.pku.edu.cn/home.do).

## Results

### Root growth determined drought tolerance properties in cotton

**Figure 1:**
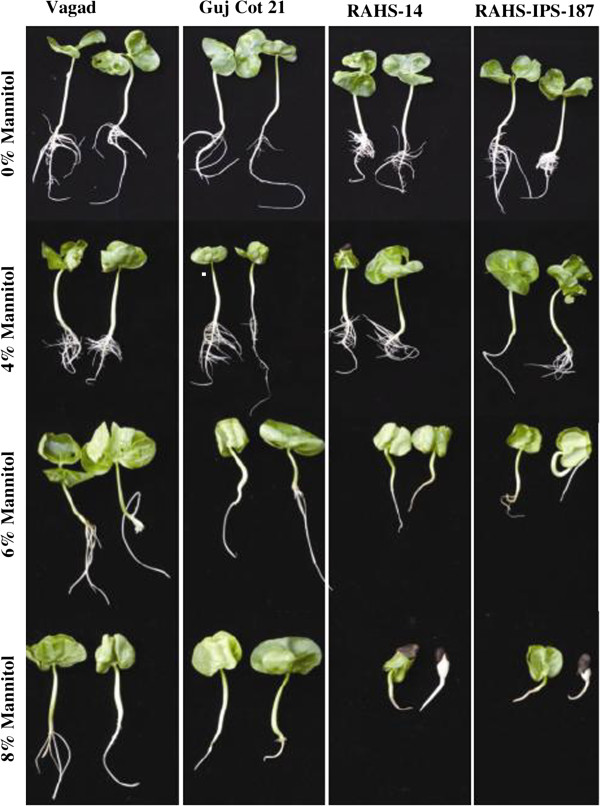
Effect of osmotic stress induced by different percentages of mannitol in the hydroponics condition on the development of the root of Vagad, GujCot21, RAHS-14, and RAHS-IPS187.

Different genotypes of *G.herbaceum* were screened for apparent differences in the root growth under control and osmotic stress conditions. The four genotypes, namely Vagad, GujCot-21, RAHS-14, and RAHS-IPS-187, exhibited contrasting differences in their root structure under control and osmotic stress conditions (Figure 1). Vagad and GujCot-21 have a longer root length as compared with RAHS-14 and RAHS-IPS-187 in the control condition. The difference was most pronounced in osmotic stress treatment by different concentrations of mannitol, which showed a significantly longer root length in Vagad and GujCot-21 at 6% of mannitol concentration as compared with a root length of RAHS-14 and RAHS-IPS-187. Further, at 8% of mannitol concentration, Vagad and GujCot-21 showed stunted growth of the root, but in RAHS-14 and RAHS-IPS-187, the development of the root was completely abolished (Figure 1). For further molecular analysis of the root of these genotypes, Vagad and RAHS-14 were analyzed by microarray, and GujCot-21 and RAHS-IPS-187 were analyzed by pyrosequencing.

### Comparative gene expression analysis of root tissue of Vagad and RAHS-14 by Affymetrix cotton gene chip

Expression profiling experiments conducted on cotton root samples from drought-tolerant (Vagad) and sensitive (RAHS-14) genotypes were compared by a commercial Affymetrix cotton gene chip, which is represented by 21,854 transcripts of cotton. The differentially expressed transcripts of the root from Vagad and RAHS-14 under drought stress and control conditions were determined. The genes that expressed equal or greater than two fold at a p-value ≤ 0.05 were assigned as differentially expressing genes for further analysis.

### Differentially expressed (DE) gene analysis

Our analysis identified 156 and 538 transcripts as being differentially up-regulated at the FC ≥ 2, p ≤ 0.05 significance level in RAHS-14 under water and drought conditions, respectively (Additional file 2 and Additional file 3). Similarly, 165 and 256 transcripts were identified as being differentially up-regulated at the FC ≥ 2, p ≤ 0.05 significance level in Vagad under watered and drought conditions, respectively (Additional file 4 and Additional file 5). Out of 538 differentially expressed genes in RAHS-14 under the drought condition, 80 genes exhibited an expression more than five fold as compared with Vagad root in the drought condition. These genes include cyclopropane fatty acid synthase, transcription regulator NOT family transcription factor, curculin-like lectin proteins, pectin esterase, metal ion transmembrane transporter, cytochrome p450, brassinosteroid insensitive 1- associated receptor kinase, and heat-shock protein family (Additional file 3). Many of the genes in RAHS-14 (probe sets 538) represent stress-responsive genes as per their GO annotation and were previously shown to be involved in abiotic stress response. Similarly, 156 genes that expressed more than two fold in RAHS-14 under the water condition include senescence-associated protein (FC > 50), zinc finger protein, dehydration-associated proteins, AP2 domains containing transcription factor, and NAC domain transcription factor observed a higher expression in RAHS-14 (Additional file 2 ). The 165 genes uniquely up-regulated in Vagad under the water condition include metal binding proteins (FC > 200), osmotin-like protein, cytochrome, and various other genes and exhibited more than two fold expression (Additional file 4). While in drought stress, the 256 up-regulated genes include Xyloglucan endotransglycosylase, serine proteases, lipid-binding protein, glycine-rich protein, sodium/potassium proton exchanger, and aquaporins proteins (Additional file 5).

### Gene ontology enrichment and Kyoto Encyclopedia of Genes and Genomes (KEGG) pathway analysis of differentially expressed genes

**Figure 2:**
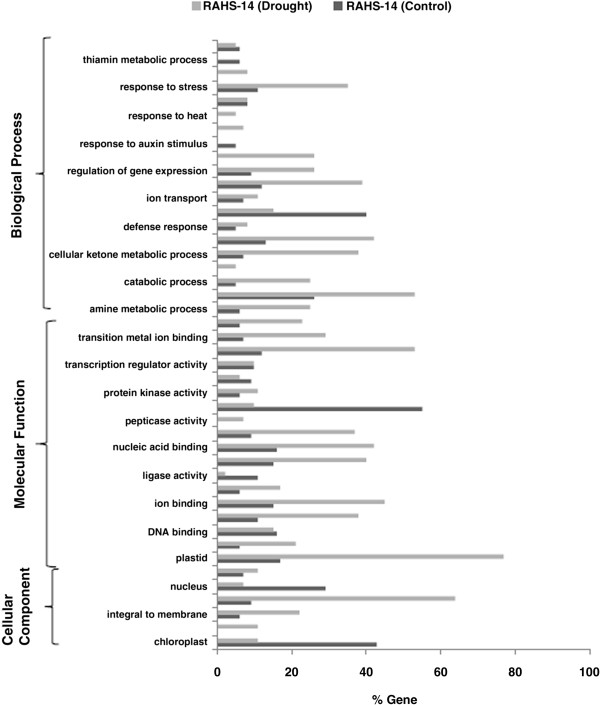
GO annotation of differentially expressed up-regulated genes in RAHS-14 during control and drought conditions.

To identify the possible biological pathways that govern the responses of differentially expressed genes, gene ontology (GO) and KEGG pathway analysis was carried out for Vagad and RAHS-14 under water and drought stress. The GO annotation mapping of RAHS-14 revealed that the genes assigned to GO terms for the thiamine metabolic process (6 genes) and response to auxin (5 genes) were exclusively enriched in the water condition (Figure 2). However, under drought stress, the genes cover a broad range of GO categories that represents response to abiotic stimulus, response to biotic stimulus, sucrose metabolic process, response to heat, cell death, and peptidase activity were enriched only under the drought condition in RAHS-14 (Figure 2). Various other stress-responsive GO terms significantly present under the drought condition were transferase activity, cellular ketone metabolic process, oxidoreductase activity, response to stress, transition metal ion binding, amine metabolic process, kinase activity, ATP binding, macromolecular complex, and integral to membrane (Figure 2). A small set of GO terms were over-represented under the water condition, such as ligase activity, protein binding, transcription factor activity, and chloroplast related genes in RAHS-14 (Figure 2). Other GO terms with a nearly similar expression level under both water and drought conditions in RAHS-14 were *response to hormone stimulus*, *transcription regulator activity*, and *DNA binding* activity in RAHS-14 (Figure 2).

**Figure 3:**
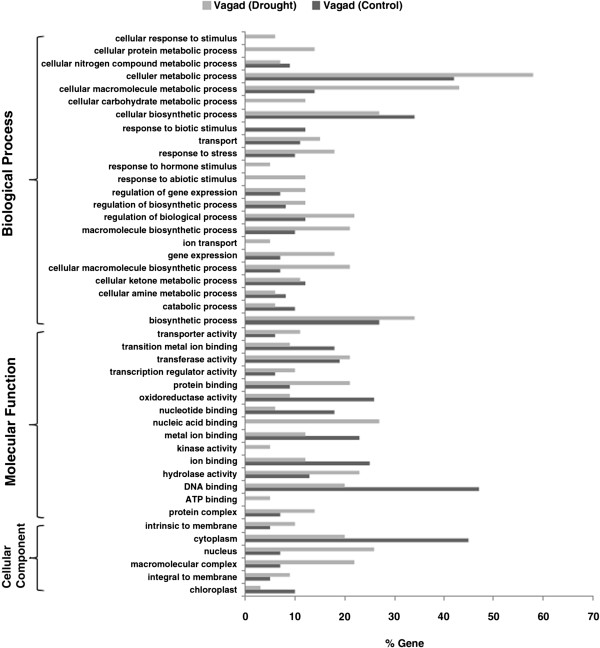
GO annotation of differentially expressed up-regulated genes in Vagad during drought and control conditions.

Similarly, GO annotation mapping in Vagad under the drought condition showed that various differentially expressed genes assigned to GO terms were involved in nucleic acid binding, response to abiotic stimulus, cellular carbohydrate metabolic process, and ATP binding (Figure 3). Likewise, the GO terms covered a broad range of stress and membrane integrity-related categories that were significantly enriched under drought as compared with the water, which includes the response to stress, hydrolase activity, macromolecular complex, regulation of the biological process, integral to membrane, intrinsic to membrane, transporter activity, cellular metabolic process, and so on (Figure 3). A single GO term *response to biotic stimulus* was present differentially under the water condition (Figure 3). Under the water condition, some interesting GO terms were over-represented in Vagad, which include *oxidoreductase activity*, *ion binding*, *metal ion binding*, *catabolic process*, *cellular amine metabolic process*, *cellular nitrogen compound metabolic process*, and so on (Figure 3). When subjected to drought stress, as an adaptative mechanism, Vagad underwent re-programming of a large number of genes; hence, almost all GO terms exhibited vast differences at the expression level.

### Microarray validation: quantitative RT-PCR analysis

**Figure 4:**
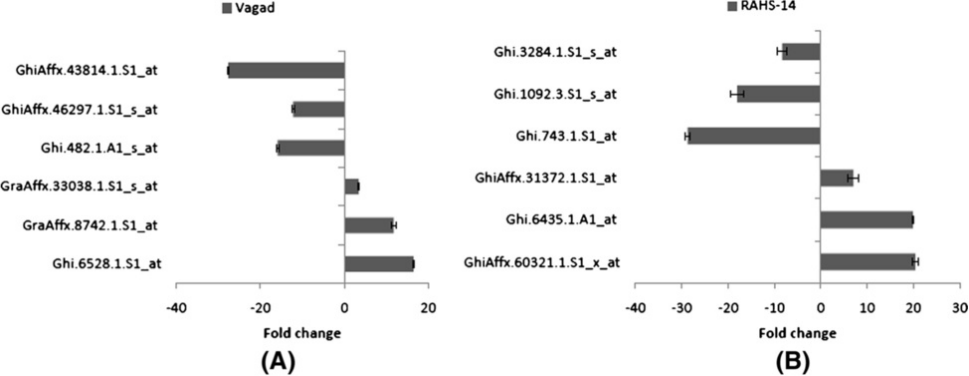
**Validation of microarray differentially expressed genes by quantitative PCR.** (**A**) Vagad (**B**) RAHS-14.

To verify the microarray results, quantitative PCR assays were carried out for the 12 selected genes. Three genes, namely Ghi.6528.1.S1_at, GraAffx.8742.1.S1_at, and GraAffx.33038.1.S1_s_at, were identified as being up-regulated in response to drought stress, and three genes, namely squalene monooxygenase (Ghi.482.1.A1_s_at), GhiAffx.46297.1.S1_s_at, and GhiAffx.43814.1.S1_at, were identified as being down-regulated in Vagad in microarray data analysis and were selected for further validation (Figure 4A). Similarly, three genes, namely GhiAffx.60321.1.S1_x_at, Ghi.6435.1.A1_at, and GhiAffx.31372.1.S1_at, were identified as being up-regulated in response to drought stress, and three genes, namely senescence-associated protein (Ghi.743.1.S1_at), Ghi.1092.3.S1_s_at, and Ghi.3284.1.S1_s_at, were identified as being down-regulated in RAHS-14 and were selected for further validation by using quantitative RT-PCR (Figure 4B). The quantitative RT-PCR analysis demonstrated that the up-regulated genes in Vagad showed 5- to 20-fold higher expressions in response to drought stress and that the down-regulated genes showed 5- to 30-fold repression in response to drought. The results were similar in case of the genes selected for RAHS-14. Thus, quantitative RT-PCR results agree with microarray analysis and, hence, validate the microarray data.

### Gene expression analysis of tolerant (GujCot-21) and sensitive genotype (RAHS-IPS-187) under drought stress by pyrosequencing of transcriptomes

Our initial analysis suggested that GujCot-21 was yet another drought-tolerant genotype, and RAHS-IPS-187 was a drought-sensitive genotype in our studies (Figure 1). Thus, the genotypes GujCot-21 (drought tolerant) and RAHS-IPS-187 (drought sensitive) of *G. herbaceum* were taken for further analysis by transcriptome sequencing of their root tissue to investigate gene expression patterns under drought stress. The 55620 and 49308 sequencing reads were obtained from GujCot-21 and RAHS-IPS-187, respectively (Table 1a). The average lengths of the assembled contigs and singletons were nearly 481 bp and 237 bp, respectively (Table 1a). The average depth of the contigs in GujCot-21 and RAHS-IPS-187 was about five reads per contig. For differential expression analysis of the genes in GujCot-21 and RAHS-IPS-187, the reads from both genotypes were tagged and pooled to form one large dataset that was assembled into contigs by using Roche’s GS-assembler (overlap length ≥40 bp and 90% identity). The 104928 reads were clustered into 2664 contigs and 50,531 singletons with an average length of 508 bp and 231 bp, respectively, for the expression analysis (Table 1a). The significant changes in gene expression as transcript per million (TPM) were calculated using the R statistics (R value ≥ 3) (Additional file 6).

**Table 1:** Summary of transcriptome sequencing data

(a)						
	** Reads**	** Bases**	** Contigs**	**Singleton**	**Av. Contig length**	**Av. S. length**
GujCot-21	55, 620	13, 020, 140	1,281	30, 501	481.7 bp	237.8 bp
RAHS-IPS 187	49, 308	11, 199, 207	858	30, 776	532.9 bp	228.6 bp
Supercontigs	1, 04, 928	24,219, 347	2, 664	50, 531	508.7 bp	231.7 bp
(b)						
	** TAIR**		** NR**		** Cotton EST**	
	Contigs	Singleton	Contigs	Singleton	Contigs	Singleton
GujCot-21	1, 011	17, 081	1, 056	18, 392	779	14,476
RAHS-IPS- 187	682	16, 856	713	9, 885	488	12, 383
Super-Contigs	2, 080	26, 838	2, 163	17204	1, 537	20, 552
(c)						
Contigs	** TAIR**	** NR**	**Cotton_EST**	**Total hits**	** No- hits**	
GujCot-21	1, 011	1, 056	779	1266	15	
RAHS-IPS-187	682	713	488	855	2	
Supercontigs	2, 080	2, 163	1, 537	2432	232	

(a) Sequencing and assembly output (b) Annotation of contigs against NCBI-NR,TAIR and cotton EST (c) total number of annotated and novel contigs (no hit).

### Annotation of root transcriptome of *Gossypium herbaceum*

The resulting contigs and singletons were queried against the NBCI NR, TAIR database for annotation using the BLASTX program at a stringency of e-value of 10E-5 and greater than 50% overlap. Each library was annotated with these databases separately. Out of the total contigs and singletons in GujCot-21, 1011 contigs and 17081 singletons were annotated with the TAIR database (Table 1b, Additional file 7), 1056 contigs and 18392 singletons with the NR database, respectively (Additional file 8). In RAHS-IPS 187, 682 contigs and 16856 singletons were annotated with TAIR, 713 contigs and 9885 singletons with the NR database, respectively (Table 1b). For the digital expression analysis, the assembled data of GujCot-21 and RAHS-IPS-187 were also annotated with these databases. Approximately 81.25% of the assembled data produced significant hits with the TAIR and NR database. A total of 2080 supercontigs and 26838 singletons were annotated with the TAIR, 2163 supercontigs and 17204 singletons with the NR, respectively. To find the common sequences between already reported cotton ESTs and our unigenes, we queried the our dataset against all publicly available cotton ESTs, at criteria of e value of 10^-5^, and at least 99% alignment of either the query or the subject. At these criteria, 779 and 488 contigs of GujCot-21 and RAHS-IPS-187, respectively, matched with cotton ESTs (Additional file 9). Only 15 sequences from GujCot-21 and 2 fromRAHS-IPS-187 did not have any match with NR, TAIR and cotton EST database and hence were considered unique genes (Table 1c). The number of contigs was assigned as unique and common in NCBI; the TAIR and cotton EST databases and presented in Additional file 10. A total of 15, 2 , and 232 contigs were uniquely present in all the 3 database of tolerant genotypes, sensitive genotypes, and supercontigs, respectively.

### Transcriptome analysis of cotton under drought stress reveals large number of drought induced genes and novel transcripts

Differentially expressed genes between GujCot-21 and RAHS-IPS187 were calculated using the R statistics (R value ≥ 3), and 2026 genes were differentially expressed in both the genotypes.For each of the contigs, the counts were converted to transcripts per million (TPM), which were transformed [log2 (fold change values)], and their differences were calculated for the fold change between GujCot-21 and RAHS-IPS-187 (Additional file 6). The quantitative profiling of the transcriptome reveals that 1503 and 1160 genes were differentially up-regulated in GujCot-21 and RAHS-IPS-187 genotypes, respectively, as compared with each other. Interestingly, 135 (17.95%) and 96 (18.17%) genes were up-regulated in GujCot-21 and RAHS-IPS-187 genotypes, respectively, and showed no hit in any database. Further, to obtain a global view of the cotton transcriptome and gene activity in two contrasting genotypes GujCot-21 and RAHS-IPS-187, differentially expressed genes were compared on the basis of their functional annotation. We found that the over-representation of the transcripts related to oxidoreductases, ribosomal proteins, membrane transporter, calcium ion binding proteins, ATPases, dehydrogenases, heat shock protein, and various enzymatic process related to other activities in GujCot21, where as in case of RAHS-IPS-187 transcripts related to hydrolases, nuclear proteins and structural proteins like structural constituent of cytoskeleton were over-represented (Additional file 6). There are several possible implications for the enrichment of these functional classes of genes in specific genotypes. The relative abundance of oxidoreductases, membrane transporter, cell wall-related proteins, and heat shock protein in GujCot-21 may be directly related to their drought-tolerant behavior.

### Functional annotation of differentially expressed genes by GO and KOBAS

**Figure 5:**
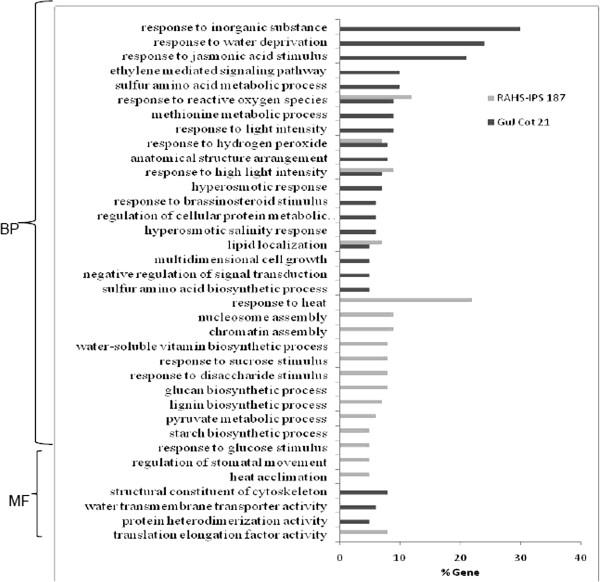
**Gene ontology of differentially expressed contigs (fold change ≥ 2) in drought-tolerant (GujCot-21) and sensitive genotypes (RAHS-IPS-187).** MF-molecular function, BP-Biological process.

**Figure 6:**
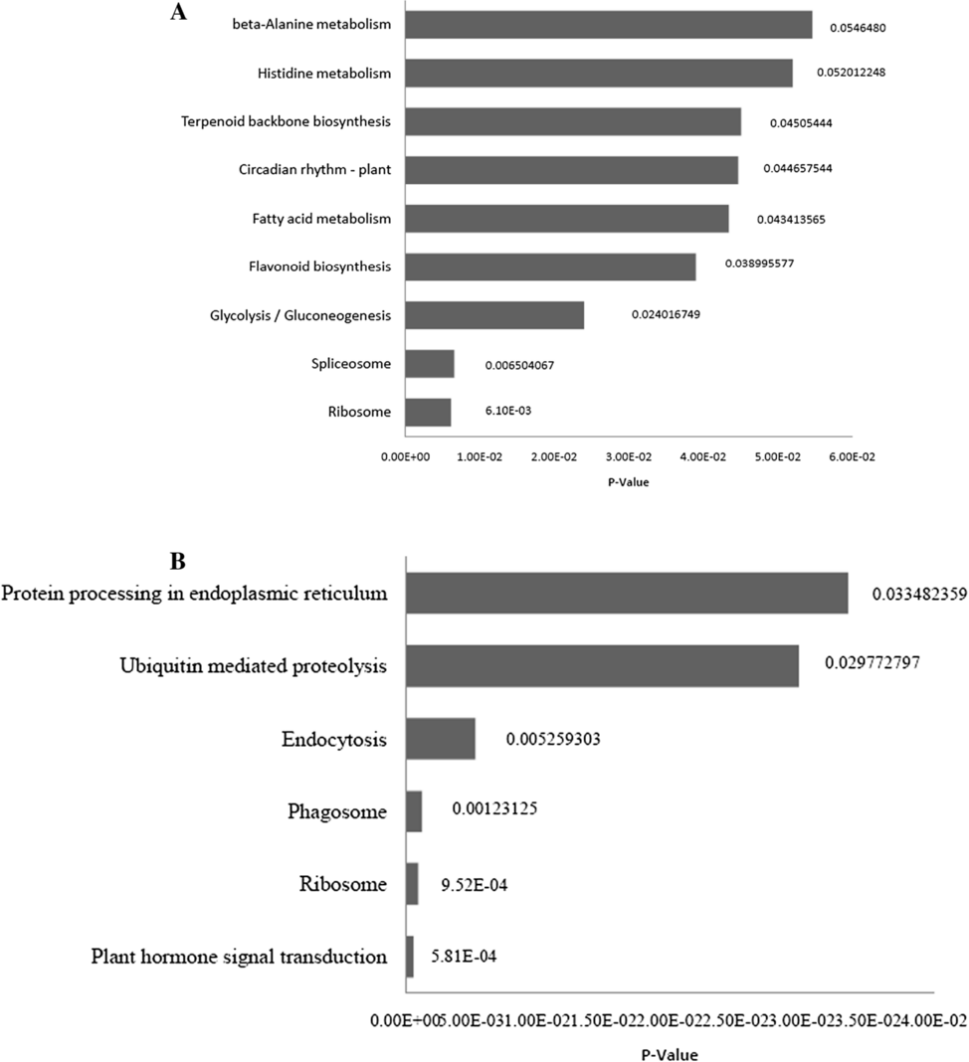
Functional enrichment of differentially expressed contigs (fold change ≥ 2) by KOBAS (A) Tolerant genotype (GujCot-21) (B) Sensitive genotype (RAHS-IPS-187).

In order to assess the differentially expressed genes with their associated biological pathways and to study the plant adaptations to limited water condition in the drought-contrasting genotype of cotton, several unique and common pathways affected by drought stress in GujCot-21 (1504 genes involved) and RAHS-IPS-187 (1161 genes involved) were identified. The significant GO terms (p-value ≤ 0.05) were characterized into biological processes and molecular functions by agriGO (http://bioinfo.cau.edu.cn/agriGO/). The most significant categories, namely response to water deprivation (24%), response to jasmonic acid stimulus (21%), ethylene-mediated signaling pathway (10%), hyperosmotic response (7%), hyperosmotic salinity response (6%), water transmembrane transporter activity (6%), and so on, were the major abiotic stress-related GO categories exclusively present in GujCot-21 and, hence, gained attention. Other GO terms exclusively present in GujCot-21 include response to inorganic substance (30%), methionine metabolic process (9%), anatomical structure arrangement (8%), response to brassinosteroid stimulus (6%), negative regulation of signal transduction (5%), and so on. Also noteworthy were the categories of response to heat (22%), water-soluble vitamin biosynthetic process (8%), response to sucrose stimulus (8%), starch biosynthetic process (5%), regulation of stomatal movement (5%), and so on, which were exclusively present in RAHS-IPS-187 with interesting GO terms. The genes associated with 4 common metabolic pathways include response to reactive oxygen species (12% and 9% of associated genes), response to hydrogen peroxide (8% and 6% of genes), response to high light intensity (7% and 9% of genes), and lipid localization (5% and 7% of genes) in GujCot-21 and RAHS-IPS-187, respectively (Figure 5 and additional file 6). In addition, the KOBAS pathway analysis was also performed to determine the various specific pathways involved in the differential up-regulation of genes. Several biochemical pathways were mapped from gene sets of GujCot-21 and RAHS-IPS-187; among them, significant KOBAS-terms were filtered with a stringent criterion of a p-value ≤ 0.05. Except one pathway, all the other KOBAS terms filtered were exclusively present in either of the genotypes. Significantly, nine KOBAS pathways were specifically modulated in GujCot-21 and six in RAHS-IPS-187 (Additional file 6). A large number of genes from GujCot-21 (91 genes) were mapped to the ribosome; ath03010, making highest a p-value of 6.10E-03, and 33 genes form RAHS-IPS-187 with a p-value of 9.52E-05. Spliceosome; ath03040 is the second-most significant in GujCot-21 and includes 17 associated genes (p-value - 0.006). Several other interesting and important pathways such as Glycolysis / Gluconeogenesis (ath00010; P-value - 0.024017), Flavonoid biosynthesis (ath00941; P-value - 0.038), Fatty acid metabolism (ath00071; P-value – 0.043), Circadian rhythm - plant (ath04712; P-value - 0.044), Terpenoid backbone biosynthesis (ath00900, P-value – 0.045), Histidine metabolism (ath00340; P-value – 0.052), and beta-alanine metabolism (ath00410; P-value – 0.054) were involved and synthesized in response to drought stress in GujCot-21 (Figure 6A). A pathway analysis of the genes present in RAHS-IPS-187 revealed plant hormone signal transduction (ath04075; P-value - 5.81E-05) to be the most significantly represented. It was followed by Phagosome (ath04145; P-value - 0.001), Endocytosis (ath04144; P-value - 0.005), Ubiquitin-mediated proteolysis (ath04120; P-value - 0.029), and Protein processing in endoplasmic reticulum (ath04141; P-value – 0.033), which were biochemical pathways affected by drought stress in RAHS-IPS-187 (Figure 6B). These biochemical pathways present in GujCot-21 and RAHS-IPS-187 indicate the complexity of the metabolic changes involved in plant responses to drought stress.

### Higher expression of antioxidant enzymes may contribute to drought tolerance in cotton

We identified the transcripts that encode various antioxidant enzymes which were predominantly expressed in GujCot-21, and, hence, these genes could be considered probable components of the complex gene networks involved in the drought condition. Hydrogen peroxide (H_2_O_2_) and superoxide are the major reactive oxygen species (ROS) that are produced in plant cells under biotic or abiotic stresses [34]. Plants have developed several strategies to minimize the oxidative damage of ROS [28]. For instance, hydrogen peroxide is eliminated by catalases (CAT) and ascorbate peroxidases (APX), and superoxide is eliminated by superoxide dismutase (SOD). Cytosolic glutathione reductases (GR) also play a role in the detoxification of H_2_O_2_[35]. Microarray differentially expressed genes (FC ≥ 2) of Vagad and RAHS-14 fetched 13 antioxidants genes, and GujCot-21 contigs from transcriptomes contributed by way of 23 antioxidant-related transcripts. The specific expression of these 36 antioxidants was further classified in three categories, namely, superoxide dismutase, peroxidase, and Glutathione S-transferase. Gujcot21 had 7 contigs encoding superoxide dismutase, 12 contigs of peroxidase, and 6 contigs related to Glutathione S-transferase. The Cu/Zn superoxide dismutase activity showed a significant 6.75-fold higher expression in GujCot-21 (contig 01875) (Table 2). Among several Glutathione S-transferases, the gene encoding GST group 8 was predominantly expressed in GujCot-21, and its activity was reported to play a major role in oxidative stress metabolism [36]. Out of 13 probe sets, 7 from RAHS-14 were distinguished into glutamate (6 probe sets) and peroxidase (1 preobe set), and the remaining 6 probe sets from Vagad were grouped into peroxidase (4 probe sets) and glutathione transferase (2 probe sets). The probe set id GhiAffx.53252.1.S1_s_at encoded glutamate synthase with highest 5.7-fold expression in RAHS-14. The 5.4-fold induction of glutathione transferase (GhiAffx.36353.1.S1_at) was highest in Vagad (Table 2). We found that most of the antioxidant enzymes were highly expressed by drought stress in GujCot-21, which implies that these antioxidant enzymes might have played important roles in plant tolerance to drought stress, whereas RAHS-IPS-187 was susceptible to drought stress.

**Table 2:** Expression level changes of various antioxidant genes under drought stress in cotton root

*Contigs represents antioxidant genes	*TPM Count/†Fold change		**Discription**
	**GUjCot-21**	**RAHS-IPS187**	
contig01875	6.753	-	Cu/Zn superoxide dismutase
contig00529	2.996	-	superoxide dismutase-like protein [Oryza sativa Japonica Group]
contig00696	2.942	-	superoxide dismutase
contig01057	2.826	-	Superoxide dismutase [Mn]; Flags: Precursor
contig01529	2.454	-	copper chaperone for superoxide dismutase [Brachypodium distachyon]
contig01075	2.411	-	Superoxide dismutase [Mn]; Flags: Precursor
contig01149	2.411	-	Cu/Zn superoxide dismutase
contig00854	2.826	-	cytosolic ascorbate peroxidase [Gossypium hirsutum]
contig00876	2.106	-	cytosolic ascorbate peroxidase 1 [Gossypium hirsutum]
contig00801	2.904	-	cytosolic ascorbate peroxidase 1 [Gossypium hirsutum]
contig01716	3.148	-	Glutathione S-transferase 8
contig02159	2.826	-	Glutathione S-transferase 8
contig01551	2.826	-	Glutathione S-transferase 8
contig02357	2.826	-	glutathione peroxidase [Populus trichocarpa]
contig01518	2.411	-	Glutathione S-transferase 8
contig00449	2.367	-	Glutathione S-transferase 8
contig00523	2.205	-	Glutathione S-transferase 8
contig01789	2.826	-	cytochrome P450 H2O2-dependent urate-degrading peroxidase [Glycine max]
contig02191	2.826	-	peroxidase [Ectocarpus siliculosus]
contig00287	2.744	-	PEROXIDASE 11
contig00850	2.563	-	cytochrome P450 H2O2-dependent urate-degrading peroxidase [Glycine max]
contig00752	2.563	-	cytochrome P450 H2O2-dependent urate-degrading peroxidase [Glycine max]
contig00393	2.411	-	putative peroxidase [Oryza sativa Japonica Group]
contig01220	2.411	-	cytochrome P450 H2O2-dependent urate-degrading peroxidase [Glycine max]
contig01737	2.411	-	PEROXIDASE 11
contig01277	2.241	-	cytochrome P450 H2O2-dependent urate-degrading peroxidase [Glycine max]
**†Probe set represents antioxidant genes**	**Vagad**	**RAHS-14**	
GhiAffx.53252.1.S1_s_at	-	5.7884912	glutamate synthase
GhiAffx.1589.38.A1_at	-	4.280462	SGT1A
GhiAffx.3996.1.S1_s_at	-	3.7647517	glutamate decarboxylase
GhiAffx.4043.6.S1_at	-	3.3622708	glutamate decarboxylase 5
GhiAffx.40354.1.S1_at	-	3.1575089	glutamate decarboxylase 5
GhiAffx.30835.1.S1_s_at	-	2.156465	Peroxidase 1
GhiAffx.22115.1.S1_x_at	-	2.1306682	Glutathione S-transferase
GhiAffx.36353.1.S1_at	5.4907985	-	glutathione transferase
GhiAffx.62078.1.S1_at	3.2424738	-	Glutaredoxin C9
GhiAffx.21573.1.S1_at	2.9193442	-	Peroxidase 10
Ghi.6564.1.S1_at	2.6120005	-	peroxidase
Ghi.6559.1.S1_at	2.31927	-	Peroxidase 73
Ghi.6746.1.A1_s_at	2.008061	-	peroxidase

### Different regulations of transporter and cell wall-related genes between the drought-tolerant and sensitive genotypes

Previous findings report that the membrane transporters, which function in cellular transport processes for the maintenance and re-establishment of homeostasis in the plant cytoplasm, were induced with various forms of abiotic stress [37]. Both RAHS-14 and Vagad showed that a large number of genes belonging to the membrane transporter family significantly increased in response to drought stress (Table 3a). However, the most contrasting difference between Vagad and RAHS-14 was that in Vagad, many membrane transporters belonging to the ABC transporter family were expressed in response to drought stress in roots. Similarly, in case of RAHS-14, the transporters belonging to the ATPase super family were predominantly expressed in response to the drought stress (Table 3a). Further, a previous study revealed that the expression of the genes involved in cell wall metabolism was generally repressed during osmotic stress in *Arabidopsis*[38]. We notably observed that 22 out of 28 genes belonging to cell wall biogenesis were repressed in both the genotypes; however, the expression of these genes was significantly higher in RAHS-14 (Table 3b). For example, the expressions of two genes (Ghi.10072.3.A1_at and GhiAffx.60321.1.S1_x_at) that putatively encode glycine-rich protein and pectinesterase were largely unaffected during drought stress in Vagad, but they were significantly down-regulated in RAHS-14 (Additional files 3 and Additional file 5). A comparative transcriptome analysis of drought-tolerant genotype (GujCot-21) and sensitive genotype (RAHS-IPS-187) also showed a similar expression pattern of the membrane transporter-related genes as had been obtained in a microarray analysis of Vagad (tolerant) and RAHS14 (sensitive). Transcriptomic analysis showed approximately 3.2% of differentially expressed genes in GujCot-21 encoding membrane transporter-related proteins. This result indicates that a comparatively less perturbation in the expression of the genes involved in cell wall biology will likely lead to better cell wall protection during drought stress in Vagad and GujCot-21 than in RAHS-14 and RAHS-IPS-187, thereby resulting in the better drought tolerance of Vagad and GujCot-21. Cell wall biogenesis plays an important role in root cell expansion and, in turn, the root architecture; therefore, we next examined in our data the genes belonging to cell wall biogenesis. We found that the number of genes responsible for cell wall biogenesis significantly increased in RAHS-14 and Vagad in response to drought stress (Table 3b). The genes representing the enzymes for cell wall biogenesis, namely cellulose synthase, glucosyl synthase, and epimerase, were found to be expressed in both Vagad and RAHS-14 in drought stress (Table 3b). However, in case of Vagad, we identified that xyloglucan: xyloglucan transferase, pectinesterase, lyases, and expansin genes were uniquely expressed in Vagad under drought stress as compared with RAHS-14. A higher expression of these genes further helps in cell wall biogenesis and, hence, probably provides better drought tolerance in Vagad.

**Table 3:** Differentially expressed genes involved in membrane transport channels and cell wall synthesis under drought stress in Vagad and RAHS-14

**(a) Membrane transporter genes family**					
**Genotypes**	**Cotton probe set ID**	**TAIR 10 hit**	**Fold change**	**p-value**	**Descriptions**
RAHS-14^D^					
	Ghi.1932.1.S1_at	AT3G46740	6.862507	0.0339456	outer membrane translocase
	Ghi.3760.1.S1_s_at	AT1G19910	2.9521239	0.02668785	ATPase, F0/V0 complex, subunit C protein
	Ghi.4450.1.A1_at	AT3G01390	2.2963412	0.01776607	V-type proton ATPase subunit G1
	Ghi.5565.6.S1_s_at	AT1G15520	2.354963	0.02798233	ABC transporter G family member 40
	Ghi.9771.1.S1_x_at	AT2G33040	3.4228158	0.02484926	Mitochondorial H + − or Na + −translocating F-type
	GhiAffx.5294.2.S1_x_at	AT2G42210	3.0888186	0.03420474	inner membrane translocas
	Gra.3081.1.A1_s_at	AT4G32530	8.158294	0.04811877	V-type and A-type ATPase (F-ATPase) Superfamily
	Gra.700.1.A1_s_at	AT1G15690	3.1466656	0.00362699	vacuolar membrane proton pump
**RAHS-14**^**C**^					
	Ghi.3763.1.A1_s_at	AT3G63380	5.7281384	0.00042	The P-type ATPase (P-ATPase) Superfamily
**Vagad**^**D**^					
	Ghi.8876.1.A1_at	AT1G51650	2.1752877	0.0222851	Mitochondorial H + − or Na + −translocating F-type
	Ghi.9195.1.A1_s_at	AT1G78720	3.98914	0.0432709	preprotein translocase Sec61 alpha subunit
	GhiAffx.13955.1.A1_at	AT3G47730	2.8928177	0.02658764	The ATP-binding Cassette (ABC) Superfamily
	GraAffx.10962.1.A1_at	AT3G47780	2.3352501	0.04103383	ABC transporter A family member 7
	GraAffx.18588.1.A1_at	AT3G59140	2.7955472	0.04233996	The ATP-binding Cassette (ABC) Superfamily
**Vagad**^**C**^					
	GraAffx.29143.1.S1_s_at	AT3G13080	2.0128014	0.0003014	The ATP-binding Cassette (ABC) Superfamily
**(b) Cell wall synthesis related gene family**					
**RAHS-14**^**D**^					
	Ghi.3798.1.A1_s_at	AT2G21770	5.2988324	0.02936104	cellulose synthase
	Ghi.6435.1.A1_at	AT5G24090	12.502564	0.02899352	acidic endochitinase (CHIB1)
	Ghi.653.1.S1_s_at	AT5G05170	2.3362005	0.04479192	cellulose synthase (Ath-B)
	Ghi.825.2.S1_x_at	AT2G40610	7.838081	0.00000635	expansin, putative (EXP8)
	GhiAffx.1589.84.S1_x_at	AT5G17310	2.6701603	0.02671632	UTP--glucose-1-phosphate uridylyltransferase
	GhiAffx.18156.1.S1_at	AT3G55250	2.8398817	0.04666388	expressed protein
	GhiAffx.36018.1.S1_at	AT4G23920	2.1763752	0.01826835	UDP-glucose 4-epimerase
	GhiAffx.46297.1.S1_s_at	AT4G17030	3.4000947	0.02354693	UTP--glucose-1-phosphate uridylyltransferase
	GhiAffx.49705.1.A1_s_at	AT1G26810	3.7181776	0.048232	galactosyltransferase family protein
	GhiAffx.52955.1.A1_at	AT4G26940	2.0691934	0.0303665	galactosyltransferase family protein
	GhiAffx.5996.1.S1_s_at	AT3G29090	3.9073741	0.02458842	galactosyltransferase family protein
	GraAffx.29883.2.S1_s_at	AT4G39210	10.707062	0.00723294	ADP-glucose pyrophosphorylase
**RAHS-14**^**C**^					
	Ghi.10493.1.S1_s_at	AT5G57560	8.634004	0.0000114	xyloglucan:xyloglucosyl transferase (TCH4)
	Ghi.1127.2.A1_x_at	AT4G25810	7.3133554	0.00000863	xyloglucan:xyloglucosyl transferase (XTR6)
	Ghi.1272.1.A1_s_at	AT2G28470	2.0890183	0.0000454	beta-galactosidase
	Ghi.4531.1.A1_at	AT4G20460	2.1103513	0.00137449	NAD-dependent epimerase
	GhiAffx.7421.1.S1_s_at	AT5G44030	50.340942	0.0000394	cellulose synthase (IRX5)
	Gra.1335.1.A1_s_at	AT5G22740	2.0989435	0.00195653	glycosyl transferase family
**Vagad**^**D**^					
	Ghi.1127.4.S1_x_at	AT4G25810	2.0596108	0.02497086	xyloglucan:xyloglucosyl transferase
	Ghi.5287.1.S1_s_at	AT4G34150	3.1211743	0.03486373	pectinesterase family protein
	Ghi.6619.1.S1_at	AT2G39700	2.451212	0.04148584	expansin, putative (EXP4)
	Ghi.6749.1.S1_s_at	AT4G38400	2.9026875	0.04047799	expansin family protein (EXPL2)
	Ghi.7660.1.S1_s_at	AT3G43270	2.7740989	0.0411485	pectate lyase family protein
	Ghi.9637.2.S1_s_at	AT3G62830	3.7921665	0.03394477	NAD-dependent epimerase/dehydratase family protein
	Gra.1335.1.A1_s_at	AT5G22740	2.9214976	0.03656408	glycosyl transferase family 2 protein
	Gra.1563.1.S1_s_at	AT2G28950	3.9881666	0.01430987	expansin, putative (EXP6)
	Gra.1984.1.A1_at	AT1G04680	4.803666	0.00806574	glycoside hydrolase family
	Gra.2243.1.A1_s_at	AT5G09760	2.1072824	0.04628986	pectate lyase family protein
	Gra.2265.1.A1_at	AT5G13870	2.4477193	0.04345882	xyloglucan:xyloglucosyl transferase (EXGT-A4)
	Gra.2300.1.A1_at	AT2G45470	4.5964513	0.03349594	pectinesterase family protein
	Gra.2380.1.A1_s_at	AT1G67750	2.7641332	0.01198629	glycoside hydrolase family
	Gra.2812.1.S1_s_at	AT4G03210	2.5062635	0.02250669	xyloglucan:xyloglucosyl transferase
	Gra.377.1.S1_s_at	AT2G06850	3.0282483	0.02482058	xyloglucan:xyloglucosyl transferase (EXGT-A1)
	GraAffx.8742.1.S1_at	AT1G32170	15.896784	0.00978865	xyloglucan:xyloglucosyl transferase (XTR4)
**Vagad**^**C**^					
	Ghi.5772.1.S1_a_at	AT4G17030	5.8524	6.0179E-07	expansin-related
	Ghi.6435.1.A1_at	AT5G24090	2.62142	1.7468E-06	acidic endochitinase (CHIB1)
	GraAffx.8742.1.S1_at	AT1G32170	3.4125	0.00012776	xyloglucan:xyloglucosyl transferase (XTR4)
	Ghi.10826.1.S1_at	AT3G57270	28.2959	2.4257E-07	glycosyl hydrolase family

RAHS-14^D^ and Vagad^D^ shows probe sets analysis under drought stress and RAHS-14^C^ and Vagad ^C^ under control condition. Cotton Probe converted unto *Arabidopis* homologous ID at criteria 50 % query match and e-value cutoff 1e-^10^.

### Transcription factors in the drought stress response in roots of *G.herbaceum*

**Figure 7:**
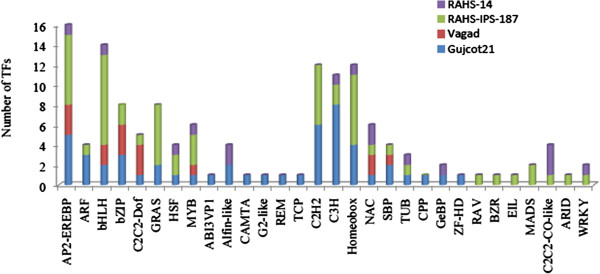
Differentially expressed TFs in all genotypes (Vagad, GujCot-21, RAHS-IPS-187 and RAHS-14).

To identify the putative transcription factors in the differentially expressed transcripts of all 4 genotypes, the cotton Affymetrix probe ID and pyrosequencing contigs were annotated with the corresponding homologous locus IDs of Arabidopsis and queried to the AGRIS database (http://arabidopsis.med.ohio-state.edu/AtTFDB/). A total of 138 genes encoding various related transcription factors belonging to 30 different families were affected under the drought condition. The genes encoding WRKY (2 genes; At2g40750; At2g23320), C2C2-CO-like (4 genes; At5g24930; At3g50410; At1g30970; and At1g43860), ARID (1 gene; At1g76510), MADS (2 gene; At3g54340; At5g48670), EIL (1 gene; At3g20770), BZR (1 gene; At1g75080), and RAV (1 gene; At1g68840) TFs were exclusively affected in sensitive genotypes (RAHS-14 and/or RAHS-IPS-187 (Figure 7). The most attention gainers were MADS, ARID, and CO-like TFs, whose related genes are associated with plant development and periodic flowering. Other TFs whose encoding genes were exclusively present in Vagad/or GujCot-21 include ZF-HD (1 gene; At1g74660), CPP (1 gene; At4g14770), TCP (1 gene; At3g27010), REM (1 gene; At4g33280), G2-like (1 gene; At4g13640), CAMTA (1 gene; At5g64220), and ABI3VP1 (1 gene; At3g18990). The ABI3VP1 has been designated as being heat and drought responsive. The CAMTA functions as a negative regulator under biotic stress and is described as being a cold responsive gene. Similarly, the induction of G2-like TF is said to confer thermo-tolerance to the plants. The bZIP (6 genes; At1g77920; At3g19290; At4g34590; At2g40950; At2g42380; and At5g28770) and ARF (3 genes; At4g23980; At5g60450; and At5g62000) were predominant in the Vagad/or GujCot-21, which are reported to be regulated by phytohormones [39,40] .There were few genes encoding various TFs whose expression was almost unanimous in all four genotype, such as MYB, NAC, and AP2/EREBP. These TFs are well reported as playing roles in developmental stages. The MYB TF is required for fiber development [41]; the NAC is a plant-specific transcription factor that is known to play a varied role in plant developmental processes [42].

### Tissue-specific expression of genes in cotton root may contribute to drought tolerance

**Figure 8:**
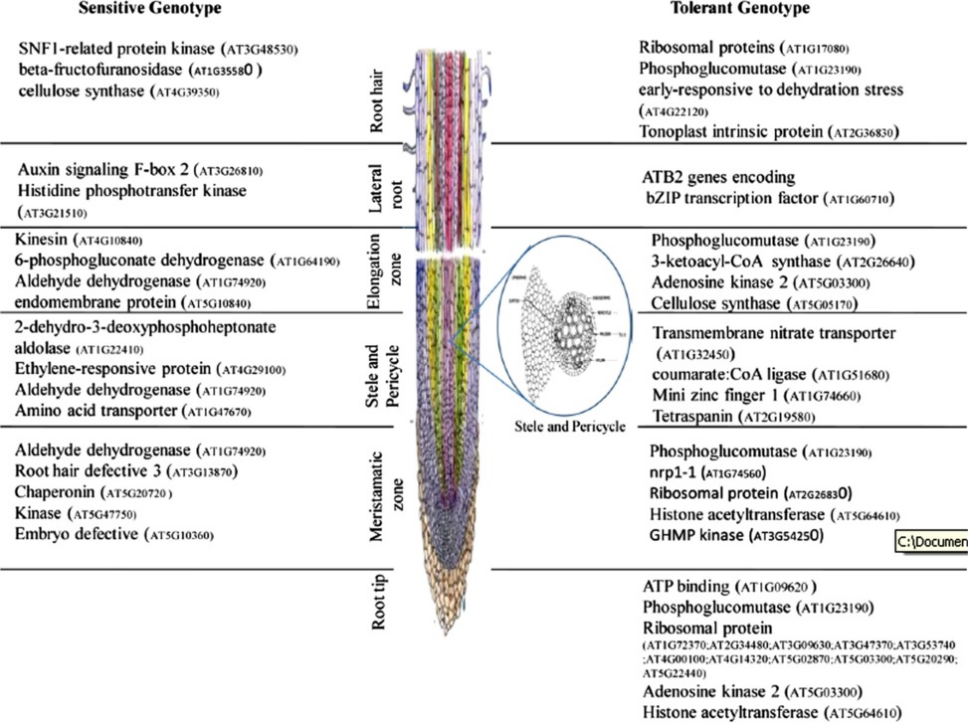
**DEGs are mapped by Genevestigator for tissue-specific expression analysis (A) tolerant and (B) sensitive genotype.** On the basis of percentage expression potential of genes in different root zones obtained from heat map were given numbers and indicated in parenthesis with their description.

**Figure 9:**
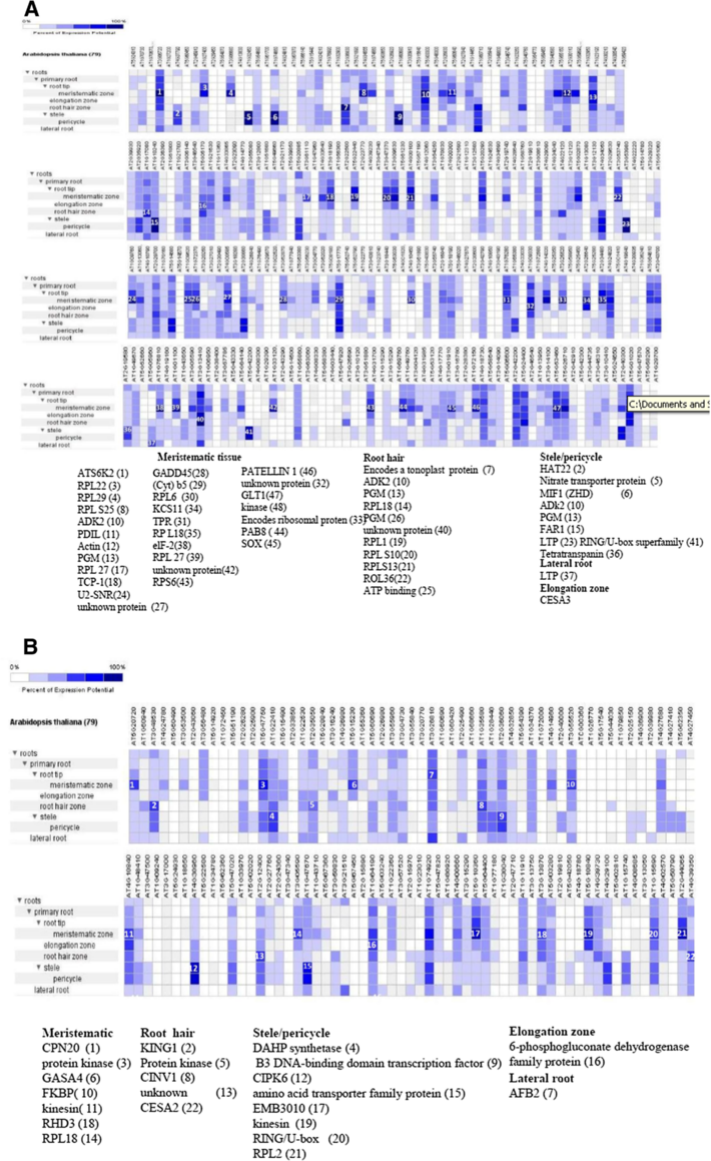
Differentially expressed genes (probe sets and contigs) analyzed by Genevestigator in mapping the specific expression of genes in different root zones.

In order to identify the tissue-specific genes in roots that respond to drought stress, differentially expressed genes in transcriptome analysis were carefully curated for their corresponding homologues in Arabidopsis, and these Arabidopsis IDs were queried against Genevestigator (Figure 8A and B) [43]. The differentially expressed genes showed specific expression in different root zones, including root hair, lateral root, elongation zone, meristamatic zone, stele, and pericycle as well as in the root tip (Figure 9, Figure 8A and B). Genevestigator results showed that the expressions of genes, such as ERD (early-responsive to dehydration stress), family protein (AT4G22120), ABRE binding factor (AT3G19290), tonoplast intrinsic protein (AT2G36830), ribosomal protein (AT1G17080), 26S proteasome (AT4G24820), methyltransferase (AT4G34050), ADH, alcohol dehydrogenase (AT5G43940), and so on, were higher in root hair of the tolerant genotype (Figure 9, Figure 8A and B); whereas in RAHS-14 genes, beta-fructofuranosidase (AT1G35580), kinases-related proteins (AT3G48530), cellulose synthase (AT4G39350), and unknown protein (AT5G02020) showed higher expression. Similarly, the lateral root zone showed the expression of only one gene that exhibited oxidoreductase activity (AT1G60710) in tolerant genotype and two genes encoding vacuolar membrane-related protein (AT3G26810) and signal transduction histidine kinase protein (AT3G21510) in sensitive genotype (Figure 9). In tolerant genotype, a greater number of genes were expressed in the elongation zone of the root as compared with sensitive genotype. The genes encoding tonoplast intisic protein (AT1G09330), cytosolic phosphoglucomutase (AT1G23190), ketoacyl-CoA synthase (AT2G26640), 26S proteasome (AT4G24820), and cellulose synthase (AT5G05170) were expressed in tolerant genotype. Similarly, the genes encoding phosphogluconate dehydrogenase (AT1G64190), aldehyde dehydrogenase (AT1G74920), and tetratricopeptide repeat (TPR)-like superfamily protein (AT4G10840) were expressed in sensitive genotype (Figure 9, Figure 8A and B). Interestingly, tolerant genotype expressed a large number of genes in meristematic, pericycle, and stele regions of the root as compared with sensitive genotype. Stele and pericycle regions of the tolerant root expressed genes such as transcription factor, FAR1-related (AT1G10240), transmembrane nitrate transporter (AT1G32450), coumarate: CoA ligase (AT1G51680), Aldo/keto reductase (AT1G60710), receptor-like protein containing leucine-rich repeats (AT1G65380), zinc finger protein (AT1G74660), and so on; whereas the genes expressed in the pericycle of sensitive genotype were related to the ethylene-responsive family protein (AT4G29100), DAHP (3-deoxy-7-phosphoheptulonate synthase) synthetase family protein, related to senescence (AT1G22410), amino-acid transporter family protein (AT1G47670), and alcohol dehydrogenase (AT1G74920) (Figure 9, Figure 8A and B). The expression analysis of the differentially expressed genes by Genevestigatotr revealed that approximately 44 and 10 genes were expressed in the meristematic tissue of tolerant and sensitive genotype, respectively. In tolerant genotype, many genes related to ribosomal family protein (AT3G06680, AT2G34480, AT3G16780, AT5G22440, AT1G27400, AT2G19740, AT3G53740, AT3G09630, AT5G02870, AT4G10450, AT3G62870, AT3G47370, AT4G33865, and AT4G34555), NAP1-related protein 1(AT1G74560), chromatin modifying enzymes (AT5G64610), and importin proteins (AT3G06720) were present. Similarly, in sensitive genotype genes related to alcohol dehydrogenase (AT1G74920), ribosomal protein (AT2G44065), calmodulin binding protein (AT5G20720), and protein kinases (AT5G47750) were highly expressed (Figure 8B). The presence of the NAP1-related transcript at a meristematic level serves as a good indicator of our root zone profiling of the differentially expressed genes. At the root tip region of the tolerant genotype, we observed the expression of the genes related to ATP binding and aminoacyl-tRNA ligases (AT1G09620), ribosomal family protein, (AT2G34480, AT3G09630, AT3G47370, and AT3G53740), and cytosolic glucose-6-phosphate dehydrogenase (AT5G40760); whereas the expression of these differential genes was absent in sensitive genotype (Figure 8A).

## Discussion

The present study reports the genome-wide expression studies conducted on the roots of drought-tolerant and sensitive genotypes of *G. herbaceum* and identifies the various genes and pathways responsible for drought tolerance. The drought-tolerant (Vagad and GujCot-21) and -sensitive genotypes (RAHS-14 and RAHS-IPS-187) showed significant variations in root structure and their length under control and mannitol stress (Figure. 1). The longer root length in Vagad and GujCot-21 genotypes showed their inherent ability to adapt to the water stress condition [44]. The adaptive advantage of increased root length and more number of root hairs that facilitate access to water under drought stress has been reported earlier [45]. Vagad and GujCot-21 displayed hydrotrophism at variable concentrations of mannitol, in which the root detects a stress gradient and directs its growth [46]. In Gujcot21 and Vagad, longer primary roots at 6% and 8% mannitol reflected an interesting plant adaptation for better survivability (Figure 1). At moderate stress of 4% mannitol, Vagad and GujCot-21 had improved anatomical adaptation by smaller leaf size and inward rolling of the leaves in order to restrict the exposed leaf surface, thereby limiting water loss by evaporation (Figure 1) [47]. The expression profiling by microarray analysis and transcriptome sequencing of the root revealed that a large number of genes such as osmotin, serine, aldo-keto reductase, LEA, chalcone synthase, chitinase, RD29, RD19, proline oxidase, ERD9, sodium hydrogen exchanger, glycin, alcohol dehydrogenase, brassinosteroids, HSP 70, and metalothionin were up-regulated in Vagad and GujCot-21 (Additional file 4, Additional file 5, and Additional file 6). Many of these genes were reported to help in osmoregulation and, thus, protect plants in stress management [4,10,29,38,48]. Many of the differentially expressed genes identified were found to represent diverse metabolic pathways (Additional file 4, Additional file 5 and Additional file 6). It was interesting to note that sensitive and tolerant genotypes underwent a larger degree of transcriptional modulation representing the various metabolic processes involved during drought stress (Figure 2, Figure 3 and Figure 5). This transcriptome modulation may eventually result in the synthesis of the secondary metabolic compounds that provide adaptive advantages to genotypes [48]. The gene ontology analysis of the differentially expressed genes obtained in microarray and transcriptome sequencing expression indicates a cross talk among various pathways that helps the tolerant genotypes to survive under drought stress. Many GO terms were exclusively present in Vagad/or Gujcot-21, and most of them were related to categories of abiotic stress, such as cellular response to stimulus, response to hormone stimulus, response to abiotic stimulus, ion transporter, kinase activity (Figure 3), response to inorganic substances, response to water deprivation, response to jasmonic acid, response to salinity, response to hyperosmotic stress, and response to salinity (Figure 5). The enrichment of these stress-related GO terms are indicative of better management of drought stress in tolerant genotypes [26]. The GO terms such as reactive oxygen species, lipid localization, lignin, sucrose, glucan, and starch biosynthesis processes were enriched in RAHS-14/or RAHS-IPS-187, but the percentages of stress-inducible GO terms and genes were less as compared with Vagad/or GujCot-21 (Figure 2 and 5). The result probably indicates that sensitive genotypes had a slow response and, hence, gradually prepared themselves to adapt to drought stress; whereas drought-tolerant genotypes were already equipped with these processes, therefore had a prompt response toward stress, and, hence, adapted better to stress. The KOBAS analysis showed significant enrichment of beta- alanine and histidine metabolism and flavanoid metabolism in GujCot-21 (Figure 6A), which indicates the formation of osmoprotective molecules under the drought condition. During drought stress, beta- alanine and histidine have been shown to increase in several different plant species, including *Arabidopsis*, maize, and wheat [49-51], and are considered as having an osmoprotective function. The secondary metabolites, such as lignin precursors, and flavonoid increase in plants under stress [52]. The presence of ABA phytohormonal pathways in RAHS-IPS-187 (Figure 6B) could be concurrent with their response to drought stress by mediating and channelizing many stress-responsive genes that help the plants in their survival over stress [53]. In contrast, Vagad and GujCot-21 adjust osmotic balance under drought stress by an increase in osmoticum deposition such as beta-alanine and catabolism, import, and utilization of various other secondary metabolites (Figure 6A, Additional file 5 and Additional file 6). The extent of osmotic adjustment in the root, although substantial, is insufficient to maintain turgor wall pressure under drought stress [48,54,55]. The transporter proteins and ion channels also play an important role in osmoregulation or drought tolerance [50]. Various membrane transporters, including ion (ABC transporters, ion channels) or sugar transporter, were enriched in Vagad under drought stress (Table 3). These transporters could both maintain the homeostasis of intracellular ion concentrations of cells and stabilize the physiological balance of plants under drought stress [29]. The higher expression of more number of genes related to membrane transporters under drought stress in tolerant genotypes (Vagad and GujCot-21) showed their plastic or adaptive behavior toward drought stress (Additional file 6, Table 3). Cell wall-loosening proteins are believed to play key roles in controlling cell wall extension [56]. The expression of expansins and xyloglucan endotransglycosylase (XET) genes were found to be higher in Vagad when drought was imposed (Additional file 5). It was reported earlier that expansin activity and extractable expansin protein increased in the root when plants were exposed to drought stress [57]. The higher expression of expansin under drought stress indicates changes in cell wall structure or the chemistry of tolerant genotypes that probably facilitate turgor pressure and the maintenance of ionic balance [56]. Genes encoding for peroxidases showed a significantly higher expression of ascorbate peroxidase, SOD in Vagad and GujCot-21 (Table 2). The expression of these oxidative radical scavenging enzymes indicates the better management of oxidative radicals in tolerant genotypes during drought stress [34]. Drought resulted in elevated expression levels of several genes in RAHS-14 and RAHS-IPS-187 like 1-aminocyclopropane-1-carboxylate oxidase (Ghi.714.1.S1_s_at; in Additional file 3 and Additional file 6), which catalyze the final step of ethylene biosynthesis [58]. Ethylene is elicited and acts as a signal mediator under almost all biotic and abiotic stress conditions, including drought [58]. The expression of ERF-related genes in sensitive genotypes under the drought condition indicates that sensitive genotypes responds to drought mainly by the ethylene pathway, leading to senescence [59]. The results were in agreement with our previous results on leaf transcriptomes for these genotypes [31]. Among TFs, the expression of the transcription factors containing the AP2-EREBP domain were higher in tolerant genotypes under drought stress (Figure 7); similarly, bHLH (basic helix loop helix) and bZIP (basic leucine zipper domain) transcription factor in Vagad and GujCot-21 were higher. TFs bHLH and bZIP belongs to a family of proteins that are specific to plants and are found to play a role in a diverse set of biological processes under biotic and abiotic stress. In case of *Oryza sativa*, the over-expression of bHLH and bZIP transcription factors resulted in the expression of several stress-responsive genes in the transgenic plants, thereby conferring the drought tolerance [53,60]. The transcription factors WRKY 75 (GhiAffx.6177.1.S1_at, GhiAffx.61976.1.S1_at) and ZAT 10 (contig00573) were exclusively expressed in Vagad/or Gujcot-21 and are known to regulate root hair development and play a role in stress conditions [17]. It was reported that RHD3 (Root hair defective 3) and LBD (Lateral organ Boundaries Domain) genes play important roles in root development and the formation of root hairs in Arabidopsis [10,60,61]. The higher expression of RHD3 and LBD genes in tolerant Vagad and GujCot-21 could be considered the key regulatory factors for better root length development and for maintaining the plasticity of roots under drought stress conditions (e.g. osmotic adjustments in roots) (Additional file 5 and Additional file 6). There was a preferential expression of genes, such as ERD (early responsive to dehydration stress) family protein, tonoplast intrinsic protein, ribosomal protein, and methyltransferase in the root hair; oxidoreductases in the lateral root region; and cellulose synthase, tonoplast intisic protein, and cytosolic phosphoglucomutase in the elongation region of the root in Vagad (Figure 8 and 8A). Cell division-related genes encoding NAP1 proteins, ribosomal proteins, and transcription factors were over-represented in the meristem. It should be noted that preferentially expressed genes in the pericycle and stele regions of the Vagad were related to nitrate transporter, coumarate-CoA ligase, and aldo/keto reductases (Figure 9 and 8A). Nitrate is the major nitrogen source for the synthesis of amino acids and nucleic acids in plants. In addition to being assimilated in the cytoplasm, it has been proposed that nitrate may be an important osmotic solute [62]. The preferential expression of the nitrate transporter in Vagad may help in the cellular homeostatic maintenance of plants under drought stress. We also observed that many genes which encode cell wall modification enzymes, such as cellulose synthase and cell expansin in the lateral root region of Vagad, are considered as playing a key role in border cell separation and the elongation of the root [55-57]. Our results showed that a significant number of genes encoding ribosomal proteins were highly expressed (fold change ≥2) in tolerant genotypes (Figure 9 and Additional file 6). Plants expressed a high number of ribosomal proteins to balance cellular protein synthesis in response to environmental variations and, at the same time, to adapt to the environment [63]. Earlier studies showed that genes encoding ribosomal proteins play an important role in the development of root meristem and contribute to the growth and development of the root system [63-65]. A higher expression of ribosomal protein could be considered important during cell growth and proliferation under drought stress in the case of tolerant genotypes. Differentially expressed genes were analyzed using the Genevestigator tool (https://www.genevestigator.com/gv/plant.jsp) (Figure 8A and B) for their specific expression in different root zones [43]. Interestingly, a large number of these genes belonged to ribosomal coding proteins and were expressed in either the meristematic zone or the root tip region, which could maintain the homeostatic balance of protein synthesis of the plant under the stress condition [66]. This comprehensive analysis of the transcriptional profile during drought stress will advance our fundamental understanding of the various genes and major metabolic pathways that provide direction for the future genetic engineering of drought-stress tolerance in cotton.

## Conclusion

The present study highlights the impact of drought on the stress-responsive signaling pathways in roots, which regulates the plant adaptation to limited water condition as well as other environmental stresses. The stress-induced transcripts had a variety of membrane transporters, such as peroxidase, catalase, WRKY, AP2 transcription factor, stress-responsive genes, and metabolic and regulatory pathways. The root transcriptome analysis revealed several important genes regulating drought tolerance and that could be considered probable potential targets for candidate gene selection in improving the tolerance of plants.

## Abbreviations

cDNA: complementary deoxyribonucleic acid; cRNA: complementary ribonucleic acid; IVT: in vitro-transcription; FC: Fold change; FDR: False detection rate; GO: Gene ontology; EST: Expressed sequence tag.

## Competing interests

The authors declare that they have no competing interests.

## Authors’ contributions

AR performed microarray and transcriptome sequencing experiments, initial data analyses and drafted the manuscript. NP performed screening of genotypes, real time PCR and analysis of microarray data. DL helps in analysis of transcriptomics data. NKD help in sample collection and other experiments. SVS mentored the entire project, designing of problem and experiments, critical discussion and suggestions. All authors read and approved the final manuscript.

## Supplementary Material

Additional file 1 List of primer used in RT-PCR Description: Word file containing list of primer sequences used for RT-PCR. Click here for file

Additional file 2 **Annotation and fold change (fold change ≥ 2) of up regulated unique genes in RAHS-14 under control condition.** Description: Excel file containing the list of unique up regulated genes and their annotation of RAHS-14 under control condition. Click here for file

Additional file 3 **Annotation and fold change (fold change ≥ 2) of up regulated unique genes in RAHS-14 under drought stress.** Description: Excel file containing the list of unique up regulated genes and their annotation of RAHS-14 under drought stress. Click here for file

Additional file 4 **Annotation and fold change (fold change ≥2) of up regulated genes (probe sets) in Vagad under control condition.** Description: Excel file containing the list of unique up regulated genes and their annotation of Vagad under control condition. Click here for file

Additional file 5 **Annotation and fold change (fold change ≥ 2) of up regulated unique genes in Vagad under drought stress.** Description: Excel file containing the list of unique up regulated genes and their annotation of Vagad under drought stress. Click here for file

Additional file 6 **Contigs analysis by R-statistics of GujCot-21 and RAHS-IPS-187 genotypes.** Description: Excel file containing detail analysis of contigs in both genotypes. Worksheet 1 containing total differential contigs, worksheet 2 is up-regulated contigs in RAHS-IPS-187, worksheet 3 is up-regulated contigs in GujCot-21, worksheet 4and 5 are agriGO of RAHS-IPS-187 and GUjCot-21 respectively. Worksheet 6 and 7 are KOBAS analysis of RAHS-IPS-187 and GUjCot-21 respectively. Click here for file

Additional file 7 **BLASTX analysis of contigs and singlets against NCBI NR database.** Description: Excel file containing BLASTX (TAIR database) results of supercontigs (worksheet1), super singleton (worksheet2), contigs of GujCot-21 (worksheet3), singletone of GujCot-21 (worksheet4), contigs of RAHS-IPS-187 (worksheet 5) and singletone of RAHS-IPS-187 (worksheet 6). Click here for file

Additional file 8 **BLASTX analysis of both transcriptome contigs and singlets against TAIR database.** Description: Excel file containing BLASTX (NCBI NR database) results of supercontigs (worksheet1), super singleton (worksheet2), contigs of GujCot-21 (worksheet3), singletone of GujCot-21 (worksheet4), contigs of RAHS-IPS-187 (worksheet 5) and singletone of RAHS-IPS-187 (worksheet 6). Click here for file

Additional file 9 **BLASTN analysis of both transcriptome contigs and singlets against publicly available cotton EST sequences.** Description: Excel file containing BLASTN (Cotton EST) results of supercontigs (worksheet1), super singleton (worksheet2), contigs of GujCot-21 (worksheet3), singletone of GujCot-21 (worksheet4), contigs of RAHS-IPS-187 (worksheet 5) and singletone of RAHS-IPS-187 (worksheet 6). Click here for file

Additional file 10 **Venn diagram of contigs obtained after BLASTX in NCBI-NR, TAIR and Cotton EST database (BLASTN).** Contigs of (A) Drought tolerant genotype (GujCot-21) (B) Sensitive genotype (RAHS-IPS-187) (C) Supercontigs Description: PPT contains a figures of venn diagram representing unique and common contigs in different database. Click here for file

## References

[B1] Bengough AG McKenzie BM Hallett PD Valentine TA Root elongation, water stress, and mechanical impedance: a review of limiting stresses and beneficial root tip traits J Exp Bot 2011 62 1 59 68 10.1093/jxb/erq350 21118824

[B2] Manavalan LP Guttikonda SK Phan Tran LS Nguyen HT Physiological and molecular approaches to improve drought resistance in soybean Plant and Cell Physiology 2009 50 7 1260 76 10.1093/pcp/pcp082 19546148

[B3] Hochholdinger F Tuberosa R Genetic and genomic dissection of maize root development and architecture Curr Opin Plant Biol 2009 12 2 172 177 10.1016/j.pbi.2008.12.002 19157956

[B4] Taramino G Sauer M Stauffer JL Jr Multani D Niu X Sakai H Hochholdinger F The maize (Zea mays L.) RTCS gene encodes a LOB domain protein that is a key regulator of embryonic seminal and post embryonic shoot borne root initiation Plant J 2007 50 4 649 659 10.1111/j.1365-313X.2007.03075.x 17425722

[B5] Courtois B Ahmadi N Khowaja F Price AH Rami JF Frouin J Hamelin C Ruiz M Rice root genetic architecture: meta-analysis from a drought QTL database Rice 2009 2 2 115 128 10.1007/s12284-009-9028-9

[B6] Henry A Gowda VRP Torres RO McNally KL Serraj R Variation in root system architecture and drought response in rice (oryza sativa): phenotyping of the OryzaSNP panel in rainfed lowland fields Field Crop Res 2011 120 2 205 214 10.1016/j.fcr.2010.10.003

[B7] Hodge A Robinson D Griffiths BS Fitter AH Why plants bother: root proliferation results in increased nitrogen capture from an organic patch when two grasses compete Plant Cell Environ 1999 22 7 811 820 10.1046/j.1365-3040.1999.00454.x

[B8] King J Gay A Sylvester Bradley R Bingham IAN Foulkes J Gregory P Robinson D Modelling cereal root systems for water and nitrogen capture: towards an economic optimum Ann Bot 2003 91 3 383 90 10.1093/aob/mcg033 12547691 PMC4244970

[B9] Lynch J Root architecture and plant productivity Plant Physiol 1995 109 1 7 13 12228579 10.1104/pp.109.1.7 PMC157559

[B10] Okushima Y Fukaki H Onoda M Theologis A Tasaka M ARF7 and ARF19 regulate lateral root formation via direct activation of LBD/ASL genes in Arabidopsis Plant Cell Online 2007 19 1 118 130 10.1105/tpc.106.047761 PMC1820965 17259263

[B11] Inukai Y Sakamoto T Ueguchi-Tanaka M Shibata Y Gomi K Umemura I Hasegawa Y Ashikari M Kitano H Matsuoka M Crown rootless1, which is essential for crown root formation in rice, is a target of an AUXIN RESPONSE FACTOR in auxin signaling Plant Cell Online 2005 17 5 1387 1396 10.1105/tpc.105.030981 PMC1091762 15829602

[B12] Hochholdinger F Park WJ Sauer M Woll K From weeds to crops: genetic analysis of root development in cereals Trends Plant Sci 2004 9 1 42 48 10.1016/j.tplants.2003.11.003 14729218

[B13] Hu J Shibata Y Zhu PP Voss C Rismanchi N Prinz WA Rapoport TA Blackstone C A class of dynamin-like GTPases involved in the generation of the tubular ER network Cell 2009 138 3 549 561 10.1016/j.cell.2009.05.025 19665976 PMC2746359

[B14] Grigg SP Galinha C Kornet N Canales C Scheres B Tsiantis M Repression of apical homeobox genes is required for embryonic root development in Arabidopsis Curr Biol 2009 19 17 1485 1490 10.1016/j.cub.2009.06.070 19646874

[B15] Galinha C Hofhuis H Luijten M Willemsen V Blilou I Heidstra R Scheres B PLETHORA proteins as dose-dependent master regulators of Arabidopsis root development Nature 2007 449 7165 1053 1057 10.1038/nature06206 17960244

[B16] Misson J Raghothama KG Jain A Jouhet J Block MA Bligny R Ortet P Creff A Somerville S Rolland N A genome-wide transcriptional analysis using Arabidopsis thaliana Affymetrix gene chips determined plant responses to phosphate deprivation Proc Natl Acad Sci U S A 2005 102 33 11934 11939 10.1073/pnas.0505266102 16085708 PMC1188001

[B17] Devaiah BN Karthikeyan AS Raghothama KG WRKY75 transcription factor is a modulator of phosphate acquisition and root development in Arabidopsis Plant Physiol 2007 143 4 1789 1801 10.1104/pp.106.093971 17322336 PMC1851818

[B18] Devaiah BN Nagarajan VK Raghothama KG Phosphate homeostasis and root development in Arabidopsis are synchronized by the zinc finger transcription factor ZAT6 Plant Physiol 2007 145 1 147 159 10.1104/pp.107.101691 17631527 PMC1976576

[B19] Chen ZH Nimmo GA Jenkins GI Nimmo HG BHLH32 modulates several biochemical and morphological processes that respond to Pi starvation in Arabidopsis Biochem J 2007 405 Pt 1 191 198 17376028 10.1042/BJ20070102 PMC1925254

[B20] Schachtman DP Goodger JQD Chemical root to shoot signaling under drought Trends Plant Sci 2008 13 6 281 287 10.1016/j.tplants.2008.04.003 18467158

[B21] Aloni R Aloni E Langhans M Ullrich CI Role of cytokinin and auxin in shaping root architecture: regulating vascular differentiation, lateral root initiation, root apical dominance and root gravitropism Ann Bot 2006 97 5 883 893 10.1093/aob/mcl027 16473866 PMC2803412

[B22] Sabatini S Beis D Wolkenfelt H Murfett J Guilfoyle T Malamy J Benfey P Leyser O Bechtold N Weisbeek P An auxin-dependent distal organizer of pattern and polarity in the Arabidopsis root Cell 1999 99 5 463 472 10.1016/S0092-8674(00)81535-4 10589675

[B23] Friml J BenkovÃ E Blilou I Wisniewska J Hamann T Ljung K Woody S Sandberg G Scheres B JÃrgens G AtPIN4 Mediates sink-driven auxin gradients and root patterning Arabidopsis Cell 2002 108 5 661 673 10.1016/S0092-8674(02)00656-6 11893337

[B24] Nishimura C Ohashi Y Sato S Kato T Tabata S Ueguchi C Histidine kinase homologs that act as cytokinin receptors possess overlapping functions in the regulation of shoot and root growth in Arabidopsis Plant Cell Online 2004 16 6 1365 1377 10.1105/tpc.021477 PMC490032 15155880

[B25] Dello Ioio R Linhares FS Scacchi E Casamitjana-Martinez E Heidstra R Costantino P Sabatini S Cytokinins determine Arabidopsis root-meristem size by controlling cell differentiation Curr Biol 2007 17 8 678 682 10.1016/j.cub.2007.02.047 17363254

[B26] Jiang Y Deyholos MK Comprehensive transcriptional profiling of NaCl-stressed Arabidopsis roots reveals novel classes of responsive genes BMC Plant Biol 2006 6 25 10.1186/1471-2229-6-25 17038189 PMC1621065

[B27] SÃnchez-FernÃndez R Fricker M Corben LB White NS Sheard N Leaver CJ Van Montagu M Inza D May MJ Cell proliferation and hair tip growth in the Arabidopsis root are under mechanistically different forms of redox control Proc Natl Acad Sci 1997 94 6 2745 2750 10.1073/pnas.94.6.2745 11038608 PMC20161

[B28] Mittler R Vanderauwera S Gollery M Van Breusegem F Reactive oxygen gene network of plants Trends Plant Sci 2004 9 10 490 498 10.1016/j.tplants.2004.08.009 15465684

[B29] Bartels D Sunkar R Drought and salt tolerance in plants Crit Rev Plant Sci 2005 24 1 23 58 10.1080/07352680590910410

[B30] Wendel JFCR Polyploidy and the evolutionary history of cotton Adv Agron 2003 78 139 186

[B31] Ranjan A Nigam D Asif MH Singh R Ranjan S Mantri S Pandey N Trivedi I Rai KM Jena SN Koul B Tuli R Pathre UV Sawant SV Genome wide expression profiling of two accession of G. herbaceum L. in response to drought BMC genomics 2012 13 1 94 10.1186/1471-2164-13-94 22424186 PMC3320563

[B32] Kulkarni VNKB Maralappanavar MS Deshapande LA Narayanan SS The worldwide gene pools of gossypium arboreum L. and G. Herbaceum L., and their improvement 2009 Springer, New York

[B33] Apel K Hirt H Reactive oxygen species: metabolism, oxidative stress, and signal transduction Annu Rev Plant Biol 2004 55 373 399 10.1146/annurev.arplant.55.031903.141701 15377225

[B34] Luna CM Pastori GM Driscoll S Groten K Bernard S Foyer CH Drought controls on H2O2 accumulation, catalase (CAT) activity and CAT gene expression in wheat J Exp Bot 2005 56 411 417 423 15569704 10.1093/jxb/eri039

[B35] Chen JH Jiang HW Hsieh EJ Chen HY Chien CT Hsieh HL Lin TP Drought and salt stress tolerance of an Arabidopsis glutathione S-transferase U17 knockout mutant are attributed to the combined effect of glutathione and abscisic acid Plant Physiol 2012 158 1 340 351 10.1104/pp.111.181875 22095046 PMC3252094

[B36] Zhang JZ Creelman RA Zhu JK From laboratory to field. Using information from Arabidopsis to engineer salt, cold, and drought tolerance in crops Plant Physiol 2004 135 2 615 621 10.1104/pp.104.040295 15173567 PMC514097

[B37] Bray EA Genes commonly regulated by water-deficit stress in Arabidopsis thaliana J Exp Bot 2004 55 407 2331 2341 10.1093/jxb/erh270 15448178

[B38] Hobo T Kowyama Y Hattori T A bZIP factor, TRAB1, interacts with VP1 and mediates abscisic acid-induced transcription Proc Natl Acad Sci 1999 96 26 15348 15353 10.1073/pnas.96.26.15348 10611387 PMC24822

[B39] Stekel DJ Git Y Falciani F The comparison of gene expression from multiple cDNA libraries Genome Res 2000 10 12 2055 2061 10.1101/gr.GR-1325RR 11116099 PMC313085

[B40] Wang S Wang JW Yu N Li CH Luo B Gou JY Wang LJ Chen XY Control of plant trichome development by a cotton fiber MYB gene Plant Cell Online 2004 16 9 2323 2334 10.1105/tpc.104.024844 PMC520936 15316114

[B41] Hu R Qi G Kong Y Kong D Gao Q Zhou G Comprehensive analysis of NAC domain transcription factor gene family in Populus trichocarpa BMC plant biology 2010 10 1 145 10.1186/1471-2229-10-145 20630103 PMC3017804

[B42] Hruz T Laule O Szabo G Wessendorp F Bleuler S Genevestigator v3: a reference expression database for the meta-analysis of transcriptomes Adv Bioinformatics 2008 2008 420747 19956698 10.1155/2008/420747 PMC2777001

[B43] Hochholdinger F Zimmermann R Conserved and diverse mechanisms in root development Curr Opin Plant Biol 2008 11 1 70 74 10.1016/j.pbi.2007.10.002 18006363

[B44] Robertson WK Hammond LC Johnson JT Boote KJ Effects of plant-water stress on root distribution of corn, soybeans, and peanuts in sandy soil Agron J 1980 72 3 548 550 10.2134/agronj1980.00021962007200030033x

[B45] Miyazawa Y Ito Y Moriwaki T Kobayashi A Fujii N Takahashi H A molecular mechanism unique to hydrotropism in roots Plant Science 2009 177 4 297 301 10.1016/j.plantsci.2009.06.009

[B46] Kadioglu A Terzi R Saruhan N Saglam A Current advances in the investigation of leaf rolling caused by biotic and abiotic stress factors Plant Science 2012 182 42 48 22118614 10.1016/j.plantsci.2011.01.013

[B47] Westgate ME Boyer JS Osmotic adjustment and the inhibition of leaf, root, stem and silk growth at low water potentials in maize Planta 1985 164 4 540 549 10.1007/BF00395973 24248230

[B48] Rampino P Pataleo S Gerardi C Mita G Perrotta C Drought stress response in wheat: physiological and molecular analysis of resistant and sensitive genotypes Plant Cell Environ 2006 29 12 2143 2152 10.1111/j.1365-3040.2006.01588.x 17081248

[B49] Yancey PH Organic osmolytes as compatible, metabolic and counteracting cytoprotectants in high osmolarity and other stresses J Exp Biol 2005 208 15 2819 2830 10.1242/jeb.01730 16043587

[B50] Wohlbach DJ Quirino BF Sussman MR Analysis of the Arabidopsis histidine kinase ATHK1 reveals a connection between vegetative osmotic stress sensing and seed maturation Plant Cell Online 2008 20 4 1101 1117 10.1105/tpc.107.055871 PMC2390728 18441212

[B51] Bohnert HJ Nelson DE Jensen RG Adaptations to environmental stresses Plant Cell 1995 7 7 1099 1111 12242400 10.1105/tpc.7.7.1099 PMC160917

[B52] Lu G Gao C Zheng X Han B Identification of OsbZIP72 as a positive regulator of ABA response and drought tolerance in rice Planta 2009 229 3 605 615 10.1007/s00425-008-0857-3 19048288

[B53] Sharp RE Hsiao TC Silk WK Growth of the maize primary root at low water potentials: II. Role of growth and deposition of hexose and potassium in osmotic adjustment Plant Physiology 1990 93 4 1337 46 10.1104/pp.93.4.1337 16667622 PMC1062677

[B54] Sharp RE Davies WJ Solute regulation and growth by roots and shoots of water-stressed maize plants Planta 1979 147 1 43 49 10.1007/BF00384589 24310893

[B55] Cosgrove DJ Loosening of plant cell walls by expansins Nature 2000 407 6802 321 326 10.1038/35030000 11014181

[B56] Wu Y Thorne ET Sharp RE Cosgrove DJ Modification of expansin transcript levels in the maize primary root at low water potentials Plant Physiol 2001 126 4 1471 1479 10.1104/pp.126.4.1471 11500546 PMC117147

[B57] Bleecker AB Kende H Ethylene: a gaseous signal molecule in plants Annu Rev Cell Dev Bio 2000 16 1 1 18 10.1146/annurev.cellbio.16.1.1 11031228

[B58] Lu PL Chen NZ An R Su Z Qi BS Ren F Chen J Wang XC A novel drought-inducible gene, ATAF1, encodes a NAC family protein that negatively regulates the expression of stress-responsive genes in Arabidopsis Plant Mol Biol 2007 63 2 289 305 17031511 10.1007/s11103-006-9089-8

[B59] Xiang Y Tang N Du H Ye H Xiong L Characterization of OsbZIP23 as a key player of the basic leucine zipper transcription factor family for conferring abscisic acid sensitivity and salinity and drought tolerance in rice Plant Physiol 2008 148 4 1938 1952 10.1104/pp.108.128199 18931143 PMC2593664

[B60] Hu Y Zhong R MorrisonIii WH Ye ZH The Arabidopsis RHD3 gene is required for cell wall biosynthesis and actin organization Planta 2003 217 6 912 921 10.1007/s00425-003-1067-7 12844267

[B61] Smirnoff N Stewart GR Nitrate assimilation and translocation by higher plants: comparative physiology and ecological consequences Physiol Plant 1985 64 2 133 140 10.1111/j.1399-3054.1985.tb02326.x

[B62] Song J Ding X Feng G Zhang F Nutritional and osmotic roles of nitrate in a euhalophyte and a xerophyte in saline conditions New Phytol 2006 171 2 357 366 10.1111/j.1469-8137.2006.01748.x 16866942

[B63] Sormani R Masclaux-Daubresse C Daniele-Vedele F Chardon F Transcriptional regulation of ribosome components are determined by stress according to cellular compartments in Arabidopsis thaliana PLoS One 2011 6 12 e28070 10.1371/journal.pone.0028070 22164228 PMC3229498

[B64] Warner JR The economics of ribosome biosynthesis in yeast Trends Biochem Sci 1999 24 11 437 440 10.1016/S0968-0004(99)01460-7 10542411

[B65] Horvath BM Magyar Z Zhang Y Hamburger AW Baka L Visser RGF Bachem CWB Bagre L EBP1 regulates organ size through cell growth and proliferation in plants EMBO J 2006 25 20 4909 4920 10.1038/sj.emboj.7601362 17024182 PMC1618091

[B66] Barakat A Szick-Miranda K Chang F Guyot R Blanc G Cooke R Delseny M Bailey-Serres J The organization of cytoplasmic ribosomal protein genes in the Arabidopsis genome Plant Physiol 2001 127 2 398 415 10.1104/pp.010265 11598216 PMC125077

