# Supplementary material for: Comparative transcriptomic analysis of roots of contrasting Gossypium herbaceum genotypes revealing adaptation to drought
Source: BMC Genomics. 2012 Nov 29;13:680. doi: 10.1186/1471-2164-13-680 (PMC3558330; doi:10.1186/1471-2164-13-680)
Supplement: Additional file 6 — Contigs analysis by R-statistics of GujCot-21 and RAHS-IPS-187 genotypes. Description: Excel file containing detail analysis of contigs in both genotypes. Worksheet 1 containing total differential contigs, worksheet 2 is up-regulated contigs in RAHS-IPS-187, worksheet 3 is up-regulated contigs in GujCot-21, worksheet 4and 5 are agriGO of RAHS-IPS-187 and GUjCot-21 respectively. Worksheet 6 and 7 are KOBAS analysis of RAHS-IPS-187 and GUjCot-21 respectively. [file 1471-2164-13-680-S6.docx]

**Additional file 10**

List of primers used for RT-PCR.

| Probe set ID | Forward primer (5’--3’ | Reverse primer (5’--3’) |
| --- | --- | --- |
| **Ghi.6528.1.S1_at** | GCATCCTTCAGCCCTACAATAGC | GGATATGAGCTTCGGAGGAAAATG |
| **GraAffx.8742.1.S1_at** | AGGCATCCCAAATGGTAGCATAT- | CGTCGATGAAGTTCCAATTCG |
| **GhiAffx.12340.1.S1_at** | ATGGCATAACAGCGAAGAAAACTG | GTTTTGGGCAATATCCGACTGAA |
| **GraAffx.33038.1.S1_s_at** | GCACAATTTCTCAACACCAGCTT | GATTCCTCGGGTTTCTGGTTTG |
| **Gra.551.1.A1_s_at** | GAAGATCCCGAGGCTTTGGAT | GATTGGGCTTCGTTGACAGTTATG |
| **Ghi.8158.1.S1_s_at** | ATGGATGCTGCACATGGTATCTT | AGAGGCGTGCCGTTGTTTC |
| **GhiAffx.60321.1.S1_x_at** | ACGCACACCTGGACATGGAA | GCGTATATTGCGTGTGTGTAACCTT |
| **Ghi.6435.1.A1_at** | TAACAAGTTCGGCAATGGTCGTA | CTTGATTCCTCGGCTTTGACAA- |
| **GhiAffx.12729.1.A1_at** | GAATCCCTCACTGCGAAAATATCC | TTCGAAACCATTCTGGTGCTACA |
| **GhiAffx.31372.1.S1_at** | CCCTCTCCCTGTCATCATCAGT- | GAGGCGCCATACTTGGTCTTAA |
