# Supplementary material for: Comparative transcriptomic analysis of roots of contrasting Gossypium herbaceum genotypes revealing adaptation to drought
Source: BMC Genomics. 2012 Nov 29;13:680. doi: 10.1186/1471-2164-13-680 (PMC3558330; doi:10.1186/1471-2164-13-680)
Supplement: Additional file 7 — BLASTX analysis of contigs and singlets against NCBI NR database. Description: Excel file containing BLASTX (TAIR database) results of supercontigs (worksheet1), super singleton (worksheet2), contigs of GujCot-21 (worksheet3), singletone of GujCot-21 (worksheet4), contigs of RAHS-IPS-187 (worksheet 5) and singletone of RAHS-IPS-187 (worksheet 6). [file 1471-2164-13-680-S7.ppt]

## Slide 1
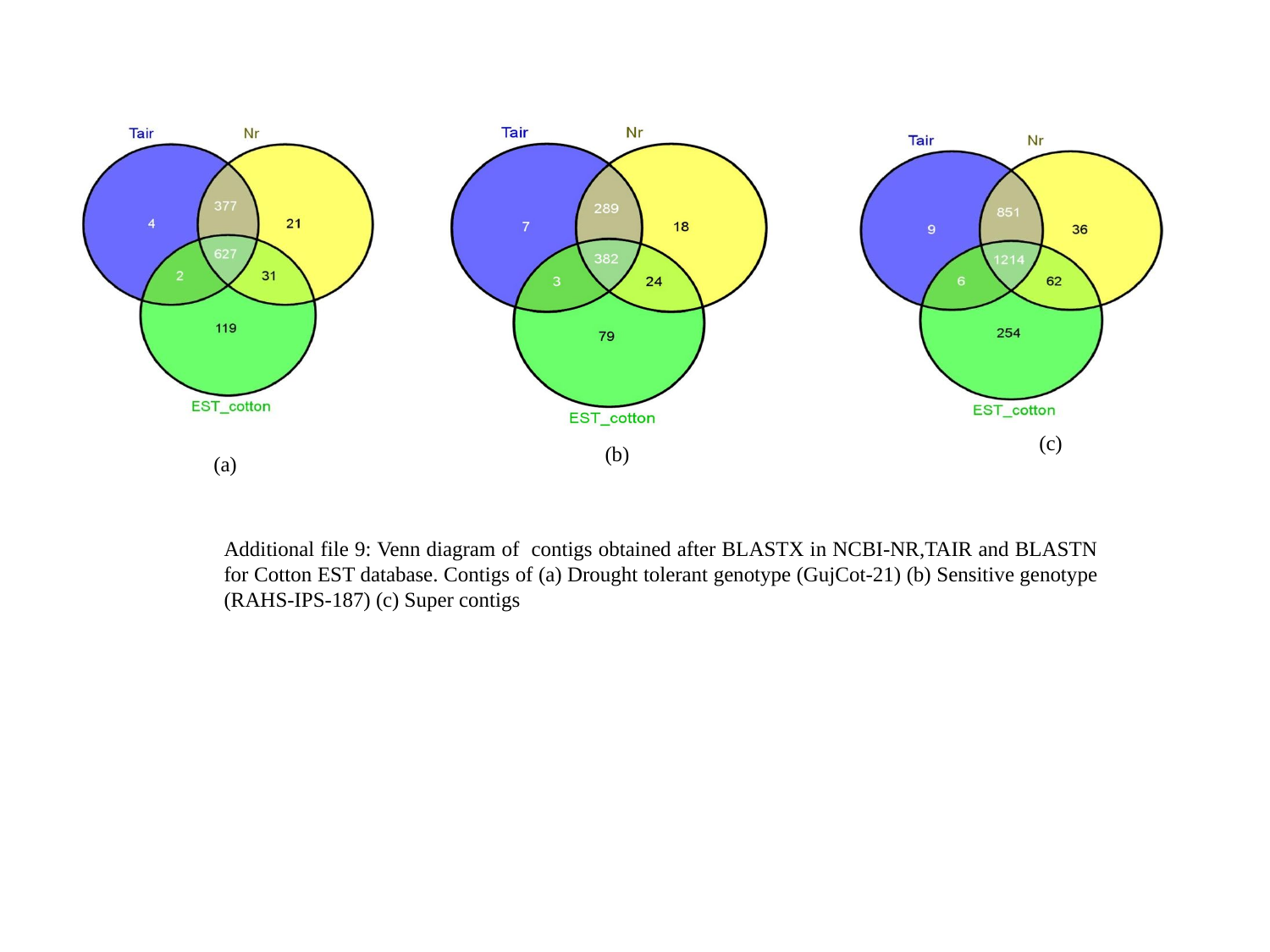

(c)
(b)
(a)
Additional file 9: Venn diagram of contigs obtained after BLASTX in NCBI-NR,TAIR and BLASTN for Cotton EST database. Contigs of (a) Drought tolerant genotype (GujCot-21) (b) Sensitive genotype (RAHS-IPS-187) (c) Super contigs
